# Projections from the dorsomedial division of the bed nucleus of the stria terminalis to hypothalamic nuclei in the mouse

**DOI:** 10.1002/cne.24988

**Published:** 2020-08-03

**Authors:** Marie Barbier, J. Antonio González, Christophe Houdayer, Denis Burdakov, Pierre‐Yves Risold, Sophie Croizier

**Affiliations:** ^1^ EA481, Neurosciences Intégratives et Cliniques, UFR Santé Université Bourgogne Franche‐Comté Besançon France; ^2^ Department of Psychiatry Seaver Autism Center for Research and Treatment, Icahn School of Medicine at Mount Sinai New York New York USA; ^3^ The Francis Crick Institute London UK; ^4^ The Rowett Institute, School of Medicine Medical Sciences and Nutrition, University of Aberdeen Aberdeen UK; ^5^ Neurobehavioural Dynamics Lab, Institute for Neuroscience, D‐HEST Swiss Federal Institute of Technology / ETH Zürich Zürich Switzerland; ^6^ University of Lausanne Center for Integrative Genomics Lausanne Switzerland

**Keywords:** AgRP/NPY, anterograde and retrograde tract tracing, dorsomedial division of the bed nucleus of the stria terminalis, hypothalamic nuclei, MCH, ORX, POMC

## Abstract

As stressful environment is a potent modulator of feeding, we seek in the present work to decipher the neuroanatomical basis for an interplay between stress and feeding behaviors. For this, we combined anterograde and retrograde tracing with immunohistochemical approaches to investigate the patterns of projections between the dorsomedial division of the bed nucleus of the stria terminalis (BNST), well connected to the amygdala, and hypothalamic structures such as the paraventricular (PVH) and dorsomedial (DMH), the arcuate (ARH) nuclei and the lateral hypothalamic areas (LHA) known to control feeding and motivated behaviors. We particularly focused our study on afferences to proopiomelanocortin (POMC), agouti‐related peptide (AgRP), melanin‐concentrating‐hormone (MCH) and orexin (ORX) neurons characteristics of the ARH and the LHA, respectively. We found light to intense innervation of all these hypothalamic nuclei. We particularly showed an innervation of POMC, AgRP, MCH and ORX neurons by the dorsomedial and dorsolateral divisions of the BNST. Therefore, these results lay the foundation for a better understanding of the neuroanatomical basis of the stress‐related feeding behaviors.

## Graphical abstract

Neurons of the dorsomedial division of the bed nucleus of the stria terminalis (BNSTdm) project onto hypothalamic nuclei including the paraventricular (PVH), dorsomedial (DMH), arcuate (ARH) nuclei and the lateral hypothalamic area (LHA). In particular, BNSTdm neurons innervate proopiomelanocortin (POMC), agouti‐related peptide (AgRP), melanin‐concentrating hormone (MCH) and orexin (ORX) expressing neurons of the ARH and the LHA, respectively.

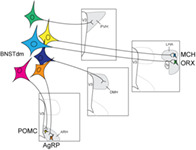

## 1 INTRODUCTION

The prevalence of obesity reached an alarming rate over the last decades. Obesity is a complex and multifactorial disease sensitive to environmental cues. Among those, daily stress plays an indisputable role in the alteration of feeding behavior and consequently in the control of body weight. In the brain, several hypothalamic nuclei are essential to control energy metabolism. The arcuate nucleus (ARH) and the lateral hypothalamic area (LHA) are ideal topics focusing most of the scientists attention as they are mainly composed of neurons involved in the tight control of energy balance and motivation (Diniz & Bittencourt, 2017; Ruud et al., 2017; Sohn, 2015; Stuber & Wise, 2016; Timper & Brüning, 2017; van der Klaauw & Farooqi, 2015). Indeed, the ARH contains the anorexigenic proopiomelanocortin (POMC)‐expressing and the orexigenic Agouti Related Peptide (AgRP)/Neuropeptide Y (NPY)‐co‐expressing neurons. For its part, the LHA is made of neuronal cells containing the melanin‐concentrating hormone (MCH) or orexin (ORX) thought to regulate feeding and reward‐related behaviors (Diniz & Bittencourt, 2017; Stuber & Wise, 2016).

Stress, anxiety and mood disorders are often associated with deregulation of feeding behavior (O'Neil et al., 2014; Weinstein et al., 1997). The interplay between psychological factors and changes in food intake during stress episode relies on telencephalic structures such as the amygdala (Ip et al., 2019). Interestingly, a few recent studies functionally linked the dorsal divisions of the anterior bed nucleus of the stria terminalis (BNST) with the LHA and the ARH neurons in controlling appetitive, aversive or exploratory behaviors (Giardino et al., 2018; González et al., 2016; Jennings et al., 2013). On the other hand, the dorsomedial division plays an indisputable role in integrating stress and anxiety behaviors (Ch'ng et al., 2018; Daniel & Rainnie, 2016; Duval et al., 2015). Indeed, the dorsomedial division of the BNST display increased number of c‐fos activated neurons upon stress exposure, and an increased dendritic arborization after chronic stress exposure (Daniel & Rainnie, 2016; Kovács et al., 2018; Vyas et al., 2003). Collectively, these data suggest an interplay between the BNST and hypothalamic neurons in the stress‐driven feeding behavior. However, BNST projections to hypothalamic nuclei have only been succinctly described without precisely compiling both the source of the projections with the downstream targets. Here, we focused our study on the projections from the dorsomedial division of the BNST on neurons of hypothalamic nuclei involved in feeding and motivated behaviors: the PVH, the dorsomedial nucleus of the hypothalamus (DMH), the ARH and the LHA with a particular interest on POMC, AgRP/NPY, MCH, and ORX neurons.

## 2 MATERIEL AND METHODS

### 2.1 Animals

#### 2.1.1 Animals used for anterograde tracing

All animal use and care protocols were in accordance with institutional guidelines and with the Directive 2010/63/EU of the European Parliament and of the Council of September 22, 2010 on the protection of animals used for scientific purposes. The protocols were approved by the Franche‐Comté University's Animal Care Committee (protocol number: 2016–0139) and the investigators were authorized. C57Bl6 male mice were obtained from Janvier (Le Genest‐Saint‐Isle, France, MGI Cat# 5752053, RRID:MGI:5752053) and were housed with a standard 12 hr light/dark cycle at a constant room temperature and had free access to the standard laboratory diet and water.

#### 2.1.2 Animals used for retrograde tracing

##### MCH and ORX neurons

All animal procedures performed in this study were approved by the UK government (Home Office) and by Institutional Animal Welfare Ethical. *Pmch*‐Cre (IMSR Cat# JAX:014099, RRID:IMSR_JAX:014099) (Kong et al., 2010) and *orexin*‐Cre (kind gift from Prof Takeshi Sakurai) (Matsuki et al., 2009) male mice were single housed and kept on a standard 12‐hr light–dark cycle (lights on at 0700 hr) and on standard mouse chow and water ad libitum.

##### POMC and AgRP neurons

All experimental procedures were approved by the Veterinary Office of Canton de Vaud (Switzerland). *Pomc*‐Cre (IMSR Cat# JAX:005965, RRID:IMSR_JAX:005965) (Balthasar et al., 2004) and *AgRP*‐IRES‐Cre (IMSR Cat# JAX:012899, RRID:IMSR_JAX:012899) (Tong et al., 2008) mice were group housed in individual cages and maintained in a temperature‐controlled room with a 12 hr light/dark cycle and provided ad libitum access to water and standard laboratory chow (Kliba Nafag).

### 2.2 Anterograde tracer into the anterior subdivisions of the BNST in the mouse

Mice were anesthetized with isoflurane (0.5 L/min) before the stereotaxic surgery. Six mice received a unilateral iontophoretic injection of 2.5% PHAL diluted in sodium phosphate buffer saline (NaPBS) pH 7.2. Glass micropipettes (tip diameter: 10–20 μm) were used to inject the PHAL iontophoretically (intermittent current of 5 μA and 7 s on/off time for 20 min). The coordinates, based on standard atlas coordinates (Franklin & Paxinos, 2008) were: AP, +0.5 mm; ML, +0.9 mm; DV, −4.0 mm. To avoid PHAL diffusion along the micropipette track, the micropipette was left in place for another 5 min before being removed. At 15 days after the injection, the brains were processed for immunohistochemistry, as described below.

### 2.3 Retrograde tracing

#### 2.3.1 MCH and orexin neurons

Data used for the present study have been generated in a previous study (González et al., 2016) where the monosynaptic retrograde tracing from MCH and ORX neurons has been extensively reported. Images have been taken from these experiments. In details, 50–60 nl of AAV2/1‐EF1a‐Flex‐C‐RVG (Addgene, 49101) and AAV2/1‐EF1a‐Flex‐eGFP‐TVA (Addgene, 26198) were stereotaxically injected into the LHA of *Pmch*‐Cre or *orexin*‐Cre mice. The injection coordinates were: 1.35 mm caudal from bregma, 0.75–0.9 mm lateral from midline, 5.3–5.4 mm ventral. Two days later we injected at the same site 70 nl of envelope A pseudotyped SADB19 rabies virus expressing tagRFP in place of rabies glycoprotein (DRV‐RFP 39) and the animal was perfused 10–11 days later. Viruses were obtained from Dr Molly Strom and were made as described in this paper (Vélez‐Fort et al., 2014).

#### 2.3.2 POMC and AgRP neurons

##### Virus

pAAV‐syn‐FLEX‐splitTVA‐EGFP‐tTA (Addgene viral prep # 100798‐AAV1; http://n2t.net/addgene: 100798; RRID: Addgene_100798) and pAAV‐TREtight‐mTagBFP2‐B19G were a gift from Ian Wickersham (Addgene viral prep # 100799‐AAV1; http://n2t.net/addgene: 100799; RRID: Addgene_100799).

##### Surgery

Monosynaptic retrograde tracing using rabies virus was performed as follow: adult 12‐week‐old *Pomc*‐Cre (Balthasar et al., 2004) and *AgRP*‐IRES‐Cre (Tong et al., 2008) mice were anesthetized with a mix of ketamine/xylazine (100 mg/kg and 20 mg/kg, respectively). Virus were injected with a microsyringe (Hamilton, 35G) and microinjection pump (World Precisions, rate at 100 nl/min). Mice receive 300 nl of mixed AAV1‐Syn‐FLEX‐splitTVA‐eGFP‐tTA and AAV1‐TREtight‐BFP2‐B19G in one side of the ARH (AP: −1.4 mm; ML: −0.3 mm; DV: −5.8 mm). After 7 days, the same mice received a second injection of 300 nl of pseudotyped rabies virus EnvA‐SADdG‐mcherry (Salk Institute) using the same coordinates. Control mice were injected with helper virus or EnvA‐SADdG‐mcherry alone. One week later, mice were anesthetized and perfused with 4% ice‐cold paraformaldehyde (PFA, Sigma) in PBS (pH 7.4) and frozen for brain cryosectioning.

### 2.4 Characterization of primary antisera

Table 1 lists the antigen, immunogen, manufacturer, catalog number, species in which the antibodies were raised, and working dilution for each of the primary antibodies. Information about the specificity of the antibodies in the following paragraphs are from the manufacturers and/or the cited references.

**TABLE 1:** Primary antibodies used in the study

Antibody	Immunogen	Source	Dilution
Anti‐PHAL	PHA‐E and PHA‐L	Rabbit, polyclonal, vector laboratories cat# AS‐2300, RRID:AB_2315142	1:1,000
Anti‐PHAL	PHA‐E and PHA‐L	Goat, polyclonal, vector laboratories cat# AS‐2224, RRID:AB_10000080	1:1,000
Anti‐TH	Denatured tyrosine hydroxylase from rat pheochromocytoma	Rabbit, polyclonal, Merck Millipore, AB152, RRID:AB_390204	1:500
Anti‐MCH	Synthetic salmon MCH; full17‐amino‐acid sequence:DTMRKMVGRVYRPCWEV	Rabbit, polyclonal (Risold et al., 1992), RRID:AB_2616562	1:1,000
Anti‐orexin A (Hcrt)	Human orexin A, immunogen AA 35–65, clone MM0500‐8G22	Mouse, monoclonal IgG1, Angio‐Proteomie, cat# hAP‐0500, ABIN1983384	1:1,000
Anti‐AVP	Human (Arg8)‐vasopressin	Rabbit, polyclonal, T‐4563, peninsula lab international, RRID:AB_518673	1:500
Anti‐NeuN	NeuN (neuron‐specific nuclear protein)	Mouse, monoclonal, Millipore cat# MAB377, RRID:AB_2298772)	1:500
Anti‐tRFP	Full‐length recombinant denatured and non‐denatured TagRFP	Rabbit, polyclonal, Evrogen cat# AB234, RRID:AB_2571743)	1:1,000
Anti‐GFP	Isolated GFP from the jellyfish *Aequorea victoria*	Chicken, polyclonal, Invitrogen, cat# A10262, RRID: AB_2534023)	1:500

The rabbit and goat polyclonal PHAL antibodies (Vector Laboratories Cat# AS‐2300, RRID: AB_2315142 and Cat# AS‐2224, RRID: AB_10000080) target both PHAL and the related *Phaseolous vulgaris* erythroagglutinin. None of them is found in the mammalian brain. The specificity was established by the absence of immunoreactivity in brain sections from naïve animals, from cases in which the uptake and transport of PHAL failed, and from regions that do not receive innervation from the area of tracer injection (Balcita‐Pedicino et al., 2011).

The rabbit polyclonal TH antibody (Merck Millipore, AB152, RRID: AB_390204) has been produced against denatured tyrosine hydroxylase from rat pheochromocytoma and targets catecholamine neurons. To test this polyclonal antibody, sections of liver has been used as negative control and brain sections (corpus striatum) and adrenal glands as positive control and produced a pattern of staining similar to that reported elsewhere in the literature (Goff et al., 2015).

The rabbit polyclonal salmon MCH (sMCH) antibody (Risold laboratory, RRID: AB_2616562) recognized the synthetic sMCH (full17‐amino‐acid, sequence: DTMRKMVGRVYRPCWEV). The specificity of the sMCH antisera was tested by blotting (Risold et al., 1992). The sMCH antibody has been tested on hypothalamic sections from many species (Chometton et al., 2014; Croizier et al., 2013). Its specificity has been verified by liquid‐phase inhibition, dot blot, and affinity column analyses (Fellmann et al., 1987; Risold et al., 1992). For mice, it was also shown that the labeling was observed exclusively in MCH‐GFP cells in the lateral hypothalamus (Croizier et al., 2011). Moreover, double labeling experiments detecting the prepro‐MCH mRNA by *in situ* hybridization and MCH peptides by indirect immunofluorescence were performed in pigs. Both signals were observed in the same cell bodies in the posterior LHA (Chometton et al., 2014).

The mouse monoclonal ORX antibody (Angio‐Proteomie, Cat# hAP‐0500, ABIN1983384) was produced from a hybridoma (mouse myeloma fused with spleen cells from a mouse immunized with a peptide, aa 35‐65, O43612) from human Orexin‐A protein. The immunohistochemistry produced a pattern of staining similar to that described elsewhere in the literature (Barbier et al., 2018).

The rabbit polyclonal AVP antibody (Peninsula Lab International, T‐4563, RRID: AB_518673) was made against synthetic human (Arg8)‐Vasopressin peptide. The immunohistochemistry highlights immunoreactive cells in the PVH and the supraoptic nucleus (SON), similar to that described elsewhere in the literature (Castillo‐Ruiz et al., 2018).

The mouse monoclonal NeuN antibody (Millipore Cat# MAB377, 140 RRID: AB_2298772) recognizes the DNA‐binding, neuron‐specific protein NeuN, which is present in most neuronal cell types of all vertebrates tested (cerebellum, cerebral cortex, hippocampus, thalamus, spinal cord and neurons in the peripheral nervous system including dorsal root ganglia, sympathetic chain ganglia and enteric ganglia). Immunoreactivity is observed in postmitotic neurons, no staining is observed in proliferative zones. The immunohistochemistry produced a pattern of staining similar to that described elsewhere in the literature (Liu & Martin, 2006).

The rabbit polyclonal tRFP antibody (Evrogen Cat# AB234, RRID: AB_2571743) was produced by using the full‐length recombinant denatured and non‐denatured TagRFP as immunogen. The antibody was made to recognize TurboRFP, TurboFP602, TurboFP635, KatushkaFP650, NirFP, TagBFP, TagRFP, FusionRed, TagFP635, mKate2, PA‐TagRFP, mRuby and mCherry. This antibody has been extensively used (González et al., 2016).

The chicken polyclonal GFP antibody (Invitrogen, Cat# A10262, RRID: AB_2534023) was produced by using GFP of jellyfish *Aequorea Victoria* as immunogen. It was used in several species (Dog, Pig, Fruit fly, Zebrafish, Human, Mouse). This antibody has been extensively used (Haehnel‐Taguchi et al., 2018).

### 2.5 Tissue preparation

Mice that received PHAL injection were deeply anesthetized with an i.p. injection of Pentobarbital (CEVA, 50 mg/kg). Animals were perfused transcardially with 0.9% NaCl, followed by ice‐cold 4% PFA (Roth) fixative in 0.1 M phosphate buffer (PB) at pH 7.4. Brains were extracted, post‐fixed for 20 hr in the same fixative at 4°C, and cryoprotected by saturation in a 15% sucrose solution (Sigma) in 0.1 M PB for 24 hr at 4°C. Tissues were cut in four series of 30 μm thick coronal sections, collected in a cryoprotective solution [1:1:2 glycerol/ethylene glycol/phosphate buffer saline (PBS)], and stored at −40°C.

Brains from *Pomc*‐Cre and *AgRP*‐IRES‐Cre mice were cut in eight series of 30 μm thick coronal sections using a cryostat and collected on Superfrost PLUS slides. They were stored at −20°C until further procedures.

### 2.6 *In situ* hybridization (RNAscope)

*Crh* mRNA detection was performed on 30 μm‐thick brain sections from 12‐week old *AgRP*‐IRES‐Cre male mice. *In situ* hybridization for *Crh* (cat # 316091) was processed using RNAscope probes and RNAscope Fluorescent Multiplex Detection Reagents (Advanced Cell Diagnostics) following manufacturer's instructions.

### 2.7 Enzymatic immunohistochemistry

After rinsing in PBS with 0.3% Triton X100 (PBS‐T), free‐floating sections were incubated with the anti‐PHAL antibodies (Table 1) in PBS‐T, 1% bovine serum albumin, 10% lactoproteins, and 0.01% sodium azide for 48 hr at 4°C. Sections were incubated for 4 hr at room temperature in a solution of biotinylated goat anti‐rabbit IgG (Table 2) at a dilution of 1:1,000 in PBS‐T. Then, sections were placed in the mixed avidin‐biotin horseradish peroxidase (HRP) complex solution (ABC Elite Kit, Vector Laboratories) for 1 hr at room temperature. The peroxidase complex was visualized by 6 min exposure to a chromogen solution containing 0.04% 3,3'diaminobenzidine tetrahydrochloride (DAB, Sigma) with 0.006% hydrogen peroxide (Sigma) in PBS. The reaction was stopped by extensive washing in PBS. Sections were then stained in a solution of 1% toluidine blue (Roth) in water to serve as a reference for cytoarchitectonic purposes. Finally, sections were dehydrated and cover‐slipped with Canada balsam (Roth).

**TABLE 2:** Secondary antibodies used in the study

Secondary antibodies	Conjugated	Manufacturer	Cat number	RRID	Dilution
Goat anti‐mouse IgG (H + L) cross‐adsorbed secondary antibody	Alexa Fluor 647	Thermo fisher	A21235	AB_141693	1:500
Goat anti‐rabbit IgG (H + L) cross‐adsorbed secondary antibody	Alexa Fluor 488	Thermo fisher	A11008	AB_143165	1:500
Goat anti‐rabbit IgG (H + L) cross‐adsorbed secondary antibody	Alexa Fluor 568	Thermo fisher	A11011	AB_143157	1:500
Donkey anti‐goat IgG (H + L) cross‐adsorbed secondary antibody	Alexa Fluor 568	Thermo fisher	A11057	AB_2534104	1:500
Goat anti‐chicken antibody IgY (H + L)	Dylight 488	Abcam	ab96947	AB_10681017	1:500
Goat anti‐rabbit IgG (H + L) cross‐adsorbed secondary antibody	Alexa Fluor 555	Thermo fisher	A21428	AB_2535849	1:500
Goat anti‐rabbit IgG	Biotinylated	Vector laboratories	BA‐1000	AB_2313606	1:1000

### 2.8 Immunofluorescent staining

After rinsing in PBS‐T, free‐floating sections were incubated with primary antibodies (Table 1) dissolved in PBS‐T, 1% bovine serum albumin, 10% lactoproteins, and 0.01% sodium azide for 48 hr at 4°C. Tissues were washed three times with PBS‐T (5 min each) and incubated for 2 hr with appropriate secondary antibodies (Table 2) diluted in PBS‐T at room temperature. Finally, sections were washed with PBS‐T, mounted using DAPI‐fluoromount (SouthernBiotech) solution.

### 2.9 Image acquisition and processing

Bright‐ and darkfield photomicrographs were acquired using an Olympus microscope BX51. Images were obtained through a DP50 numeric camera (Olympus, France) using the AnalySIS software. Immunofluorescent sections were acquired on a confocal LSM 710 (Zeiss, Germany) equipped with lasers for excitation of Alexa 488 (488 nm), Alexa 568 (561 nm), Alexa 647 (633 nm) and DAPI (405 nm) and Plan Apochromat 10X 0.45 DIC, Plan Apochromat 20X 0.8 DIC and Plan Apochromat 63X Oil DIC. Images were obtained by using the Zen black 2012 software. For imaging details regarding the retrograde tracing study (MCH and ORX) see González et al., 2016. Images taken for the retrograde tracing study (POMC and AgRP) were acquired on a ZEISS Axio Imager.M2 microscope, equipped with ApoTome.2 and a Camera Axiocam 702 mono (Zeiss, Germany). Specific filter cubes were used for the visualization of green (Filter set 38 HE eGFP shift free (E) EX BP 470/40, BS FT 495, EM BP 525/50), red (Filter set 43 HE Cy 3 shift free (E) EX BP 550/25, BS FT 570, EM BP 605/70), and blue (Filter set 49 DAPI shift free (E) EX G 365, BS FT 395, EM BP 445/50) fluorescence. Different magnifications were selected using a Zeiss ×10 objective (Objective Plan‐Neofluar ×10/0.30, M27, FWD = 5.2 mm) and a x20 objective (Objective Plan‐Apochromat ×20/0.8 M27, FWD = 0.55 mm).

**FIGURE 1:**
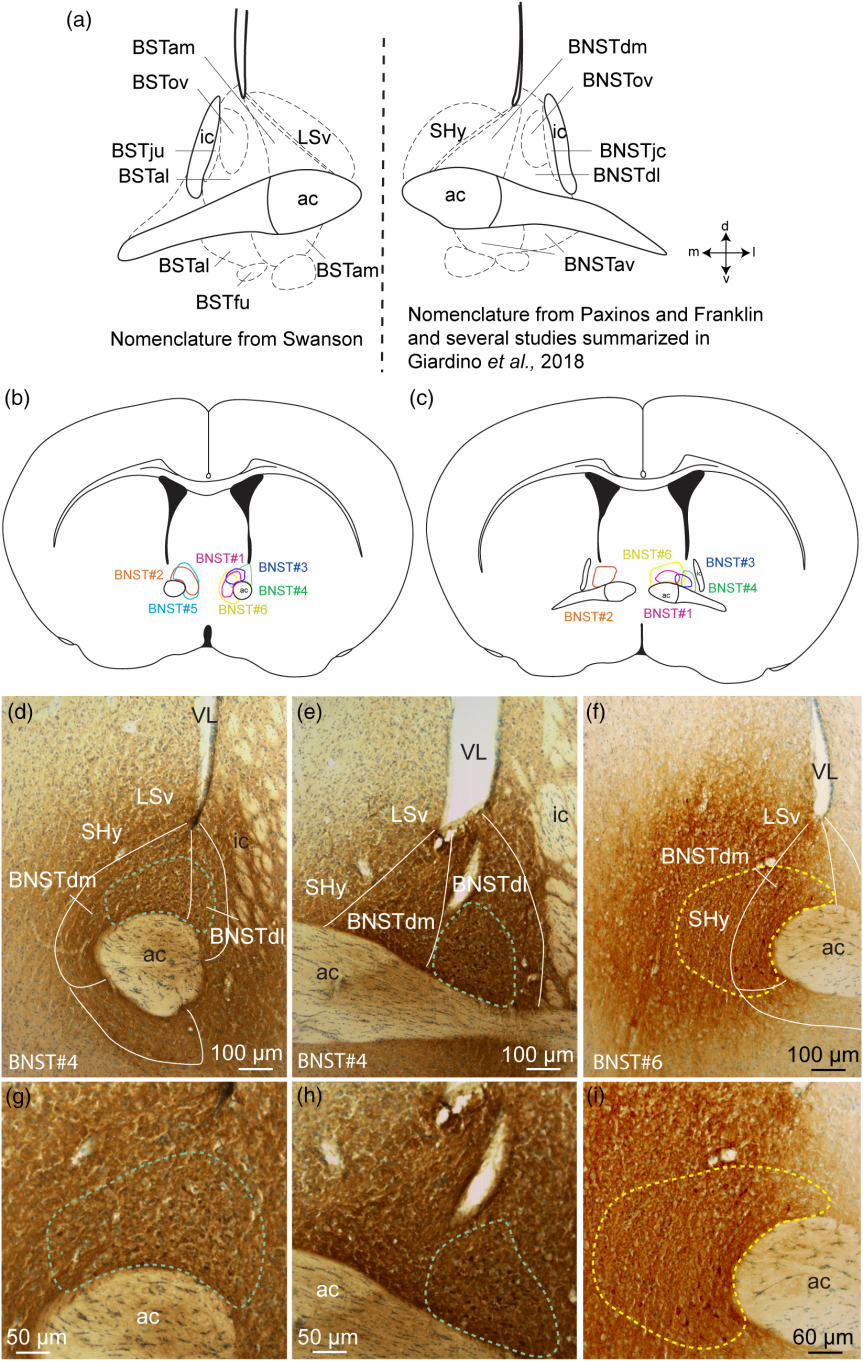
(a) Schematic illustration of the divisions of the bed nucleus of the stria terminalis (BST) based on Swanson (left) and (BNST) Paxinos and Franklin nomenclatures and others summarized in Giardino et al,. (2018) (right). (b, c) Line drawings to illustrate the localization of the six PHAL injection sites into the dorsomedial and dorsolateral divisions of BNST. (d–i) Photomicrographs illustrating the injection sites in experiments BNST#4 and BNST#6. ac, anterior commissure; BSTal, BST anterior division, anterolateral area; BSTam, BST anterior division, anteromedial area; BNSTav, anteroventral division of the BNST; BNSTdm, dorsomedial division of the anterior BNST; BNSTdl, dorsolateral division of the anterior BNST; BSTfu, BST anterior division, fusiform nucleus; BNSTjc, juxtacapsular nucleus of the BNST; BSTju, BST anterior division, juxtacapsular nucleus; BSTov, BST anterior division, oval nucleus; BNSTov, oval nucleus of the BNST; ic, internal capsule; LSv, ventral part of the lateral septum; SHy, septohypothalamic nucleus; VL, lateral ventricle [Color figure can be viewed at wileyonlinelibrary.com]

Neither additional treatment was made, except to enhance fluorescent intensity. Nomenclature and nuclear parcellation are based on Mouse Brain Atlas from Franklin and Paxinos (Franklin & Paxinos, 2008), on Rat Brain Atlas from Swanson (Swanson, 2004) and on other studies summarized in Giardino et al., 2018 (Figure 1a).

### 2.10 Analysis of PHAL fibers density

For the quantitative analysis of PHAL fibers density, each image plane was binarized using Image J to isolate labeled fibers from the background and to compensate for differences in fluorescence intensity. A 220 × 220 μm region of interest (ROI) was placed over the structures of interest. The integrated intensity, which reflects the total number of pixels in the binarized image, was then calculated for each image. The PHAL fibers density calculated in the LHA after injection in the BNSTdm (BNST#2) or in the septohypothalamic nucleus (SHy, BNST#6) was defined as 100%. Relative PHAL fibers density was thus calculated for the PVH, DMH, LHA, VMH, ARHd (dorsal) and ARHvm (ventromedian).

## 3 RESULTS

### 3.1 PHAL injection sites onto the dorsomedial division of the BNST

Six experiments were analyzed to depict the projections from the dorsomedial division of the BNST to hypothalamic areas (Figure 1b,c). For two of them (BNST#1, pink and BNST#2, orange, Figure 1b,c) the PHAL injection sites were exclusively restricted to the dorsomedial division of the BNST (Bregma +0.50–0.26 of the Franklin and Paxinos Mouse Atlas, 2008). Two additional experiments (BNST#3, blue and BNST#4, green, Figure 1b–e,g,h) were centered in the dorsomedial division of the BNST (Bregma +0.50–0.38 of the Franklin and Paxinos Mouse Atlas, 2008) and slightly involved the dorsolateral part of the BNST (Bregma +0.26 of the Franklin and Paxinos Mouse Atlas, 2008) (Figure 1b,c). The last two PHAL injection sites involved both the dorsomedial and ventromedial parts of the BNST (Bregma +0.50–0.26 of the Franklin and Paxinos Mouse Atlas, 2008, BNST#5, cyan, Figure 1b,c) with in addition a contamination of the medial part of the septohypothalamic nucleus for one of them (Bregma +0.26 of the Franklin and Paxinos Mouse Atlas, 2008, BNST#6, yellow, Figure 1b,c,f,i).

### 3.2 General distribution patterns of PHAL axons

**FIGURE 2:**
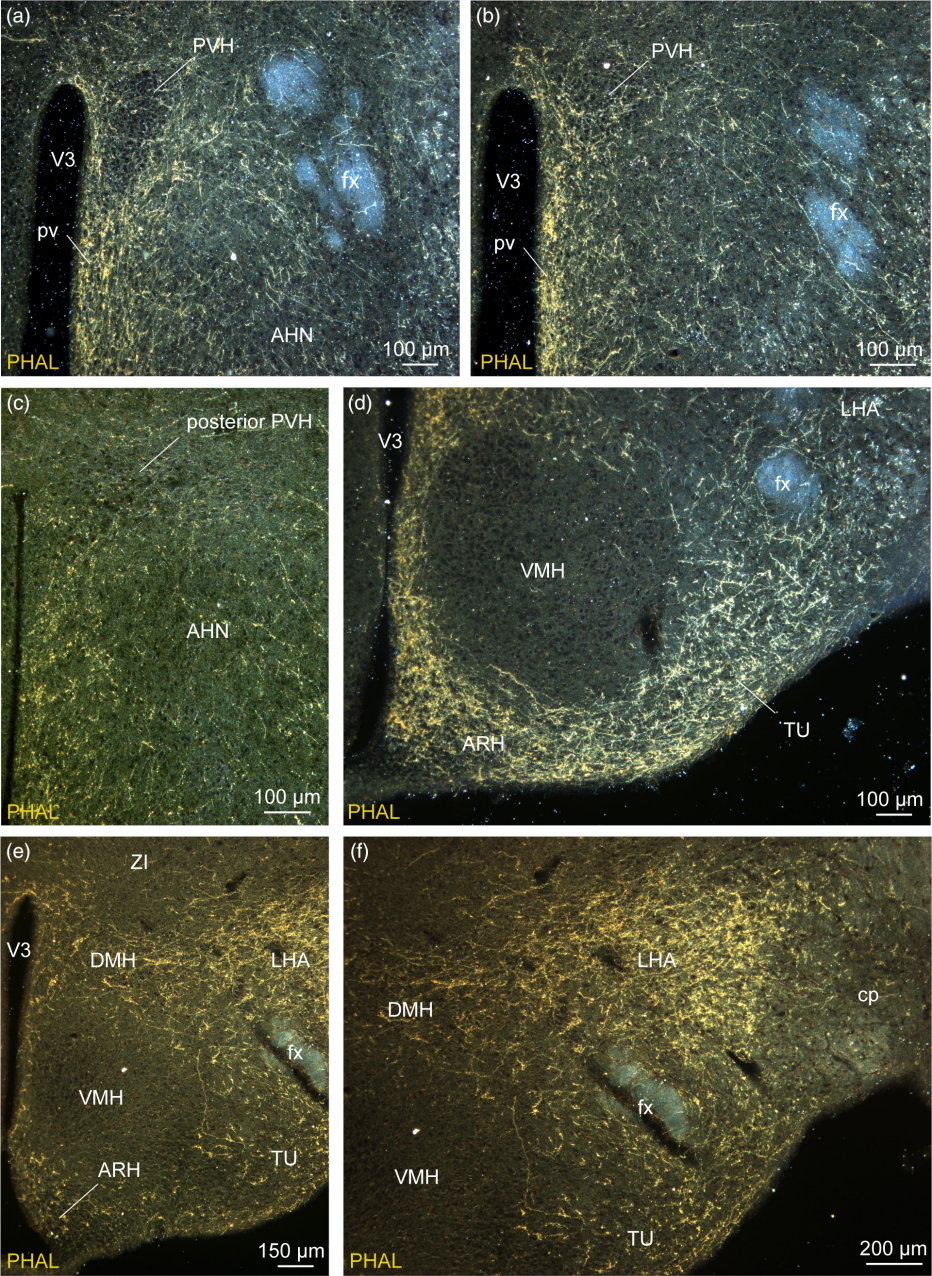
Darkfield photomicrographs illustrating the distribution of PHAL axons from the dorsomedial BNST in the pv (a, b), in the PVH (a–c), in the VMH (d–f), in the LHA (d–f), in the ARH (d, e) and in the tuberal nucleus (d, e). These areas receive light (anterior hypothalamic nucleus, AHN), moderate (PVH) to intense (LHA) innervation from the anterior divisions of the bed nucleus of the stria terminalis. Three experiments are illustrated: Experiment BNST#6 (a, b, d), experiment BNST#1 (c) and experiment BNST#4 (e, f). ARH, arcuate nucleus of the hypothalamus; cp, cerebral peduncle; DMH, dorsomedial nucleus of the hypothalamus; fx, fornix; LHA, lateral hypothalamic area; pv, periventricular nucleus; PVH, paraventricular nucleus of the hypothalamus; TU, tuberal nucleus; VMH, ventromedial nucleus of the hypothalamus; V3, third ventricle; ZI, zona incerta. Scale bars are shown in the figure [Color figure can be viewed at wileyonlinelibrary.com]

**FIGURE 3:**
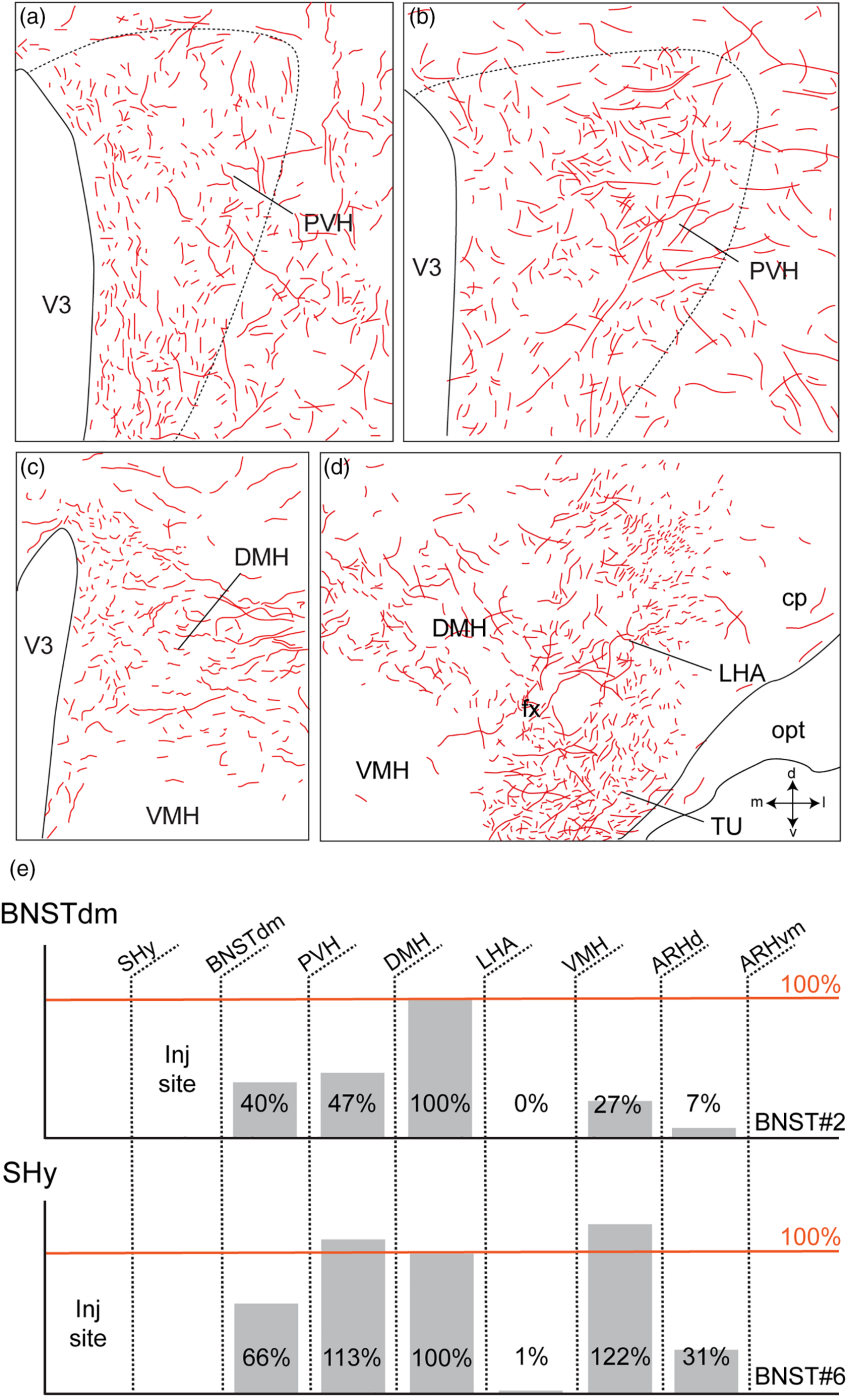
Computerized cartographies of the anterodorsal division of PHAL‐labeled axons (red) from the dorsomedial BNST in the PVH (a, b), in the DMH (c, d) and in the LHA (d) based on camera Lucida method. Long parallel axons coming from the LHA are observed in the lateral division of the DMH (c). Drawings in d highlights intense innervation of the TU and the LHA. Crossed arrows (d) indicate the orientation of the drawings (m, medial; l, lateral; d, dorsal and v, ventral). (e) Quantification of the PHAL fibers density in the PVH, DMH, VMH, LHA, ARHd (dorsal), and ARHvm (ventromedial) relative to the density measured in the LHA (defined as 100%) after injection in the dorsomedial division of bed nucleus of the stria terminalis (BNSTdm, BNST#2) or in the septohypothalamic nucleus (SHy, BNST#6). Cp, cerebral peduncle; DMH, dorsomedial nucleus of the hypothalamus; fx, fornix; LHA, lateral hypothalamic area; opt, optic tract; TU, tuberal nucleus; VMH, ventromedial nucleus of the hypothalamus; V3, third ventricle [Color figure can be viewed at wileyonlinelibrary.com]

In all experiments, the PVH in the anterior hypothalamus, and most nuclei of the tuberal hypothalamus including the DMH, the LHA and the ARH received light to intense inputs (Figures 2 and 3). By contrast, the VMH was entirely surrounded by these projections, but contained very few axons (Figure 2d,e).

In the experiments centered in the dorsomedial division of the BNST, PHAL‐positive axons were mainly observed in periventricular nuclei such as the PVH, the DMH and the ARH (Figure 2a–e). In the experiment BNST#6, the injection of PHAL targeted the dorsomedial division of the BNST with a medial contamination in the adjacent septohypothalamic nucleus, PHAL‐labeled axons provided a more intense innervation in the ARH and a lower number of PHAL‐positive fibers in the LHA (Figures 2d and 3e). On the contrary, the injection site of the experiment BNST#4 involving the dorsomedial and dorsolateral BNST provided a moderately intense PHAL‐innervation in the dorsolateral ARH while a high intensity of PHAL‐positive fibers was observed in the LHA (Figures 2e,f and 3e).

The overall and specific innervation by the dorsomedial division of the BNST PHAL‐positive fibers onto the aforementioned nuclei and characteristic neuronal populations will be detailed in the following paragraphs.

### 3.3 Pattern of innervation of the periventricular nuclei

In mice, the PVH is poorly compartmentalized when compared to that described in rats (Biag et al., 2012; Simmons & Swanson, 2009). In the neuroendocrine division, it is nearly impossible to distinguish magnocellular from parvicellular divisions only based on a Nissl stain (Figure 3a,c). The distribution of magnocellular (Arginine Vasopressin, AVP; Oxytocin, OXT) and parvicellular (Corticotropin‐releasing hormone, CRH; Thyrotropin‐releasing hormone, TRH; Somatostatin, SST; Tyrosine hydroxylase, TH) neurons are not as segregated as in rats (Biag et al., 2012). In this study, we did not intend to precisely delineate PVH divisions in mice, but we based our descriptive and structural analyses on published data, on immunostainings and *in situ* hybridization for known PVH markers such as AVP, TH and *Crh*.

**FIGURE 4:**
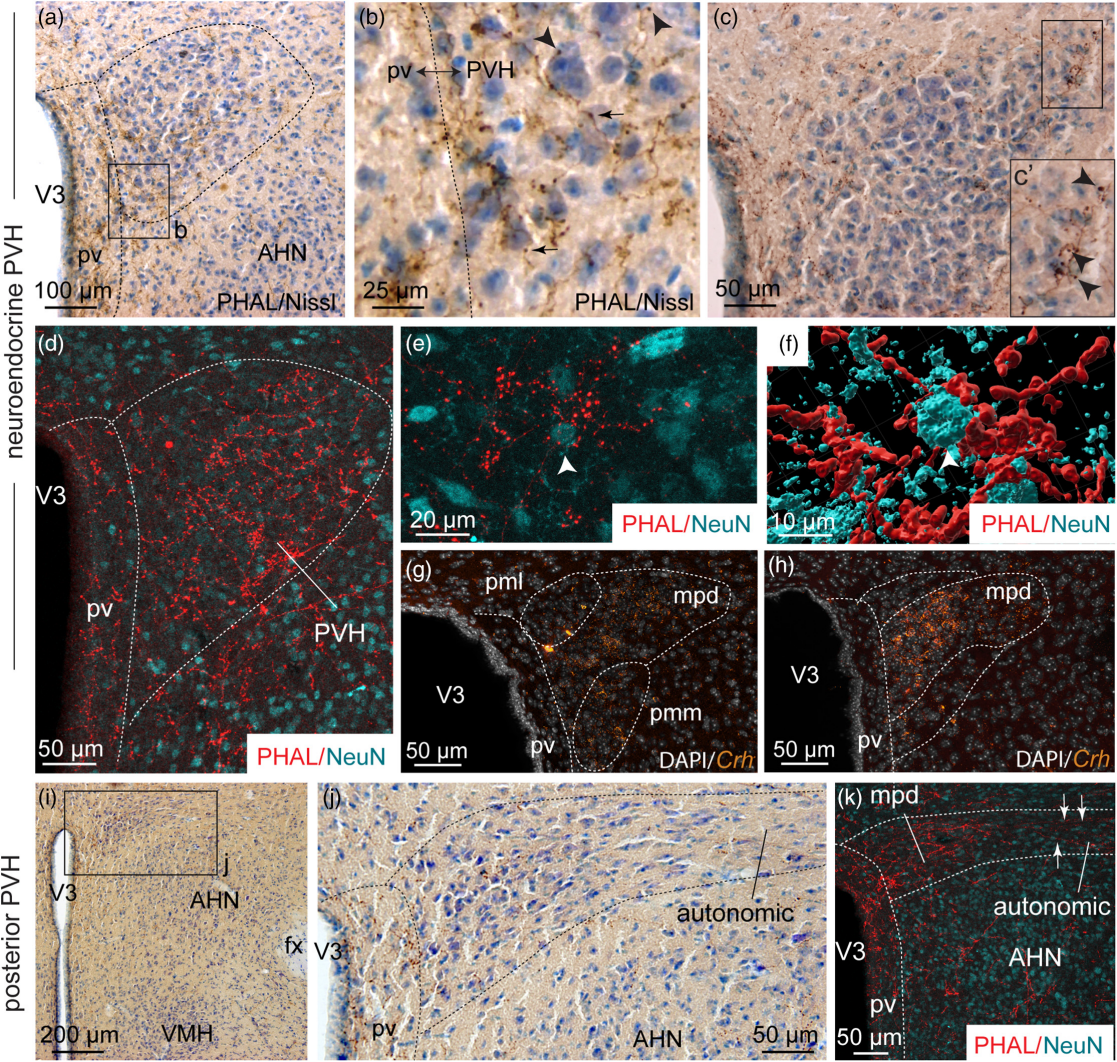
(a) Photomicrograph showing Nissl‐stained sections for cytoarchitectonic purposes of the PVH combined with enzymatic detection of PHAL from the dorsomedial BNST (experiment BNST#6). (b) High magnification of photomicrograph illustrating varicosities (arrows) and short collaterals ended by terminal boutons (arrowheads) in the neuroendocrine part of the PVH. (c) Photomicrograph illustrating PHAL from the dorsomedial BNST and Nissl‐stained sections in the neuroendocrine PVH. (c′) High magnification illustrating a long PHAL‐positive axon that displays numerous terminal boutons (arrowheads) close to nucleated cells labeled with Nissl in the lateral extremity of the neuroendocrine PVH. (d) Photomicrograph showing double immunodetection of PHAL‐positive fibers (red) from the dorsomedial BNST and neurons labeled with NeuN (cyan) in the neuroendocrine part of the PVH and the periventricular nucleus (pv) (experiment BNST#2). (e) High magnification of PHAL‐positive fibers (red) from the dorsomedial BNST in the vicinity of a neuron labeled with NeuN (cyan) in the neuroendocrine PVH (experiment BNST#2). (f) 3D reconstruction using Imaris software of PHAL‐positive fibers (red) from the dorsomedial BNST in contact with a PVH neuron (white arrow, cyan). (g, h) Photomicrographs showing *Crh* mRNA‐expressing cells (orange) and DAPI counterstaining (white) at two distinct levels of the neuroendocrine PVH. Low (i) and high magnifications (j) of PHAL and Nissl‐stained sections in the posterior part of the PVH (experiment BNST#6). (k) Photomicrograph showing double immunodetection of PHAL‐positive fibers (red) from the dorsomedial BNST and neurons labeled with NeuN (cyan) in the posterior part of the PVH (experiment BNST#2). The autonomic part of the PVH is observed in the lateral part of the PVH while the medial part contains the parvicellular neurons. AHN, anterior nucleus of the hypothalamus; fx, fornix; pv, periventricular nucleus; mpd, medial parvicellular part, dorsal zone of the PVH; pml, posterior magnocellular part, lateral zone of the PVH; pmm, posterior magnocellular part, medial zone of the PVH; PVH, paraventricular nucleus of the hypothalamus; VMH, ventromedial nucleus of the hypothalamus; V3, third ventricle. Scale bars are shown in the figure [Color figure can be viewed at wileyonlinelibrary.com]

Dorsomedial BNST axons provided light to moderate inputs to the neuroendocrine and autonomic divisions of the PVH (Figures 2a–c, 3a,b, and 4a–d). Numerous varicosities and a few boutons terminating short collaterals in contact with Nissl‐stained cells suggested axosomatic synaptic contacts (Swanson, 2004) in the neuroendocrine parts of the rostral and caudal PVH (Figure 4b,c,c′,j). A dual immunolabeling with NeuN and 3D reconstruction clearly illustrated that some neurons were targeted by multiple synaptic boutons (Figure 4e,f). In mice, *Crh*‐containing neurons are mostly observed in PVH parvicellular division (PVHmpd) like in rats (Figures 3b and 4g,h) (Biag et al., 2012). However, PHAL detection was not performed on the sections used to label *Crh* and whether PHAL‐positive terminals specifically targeted CRH neurons will require further analyses.

**FIGURE 5:**
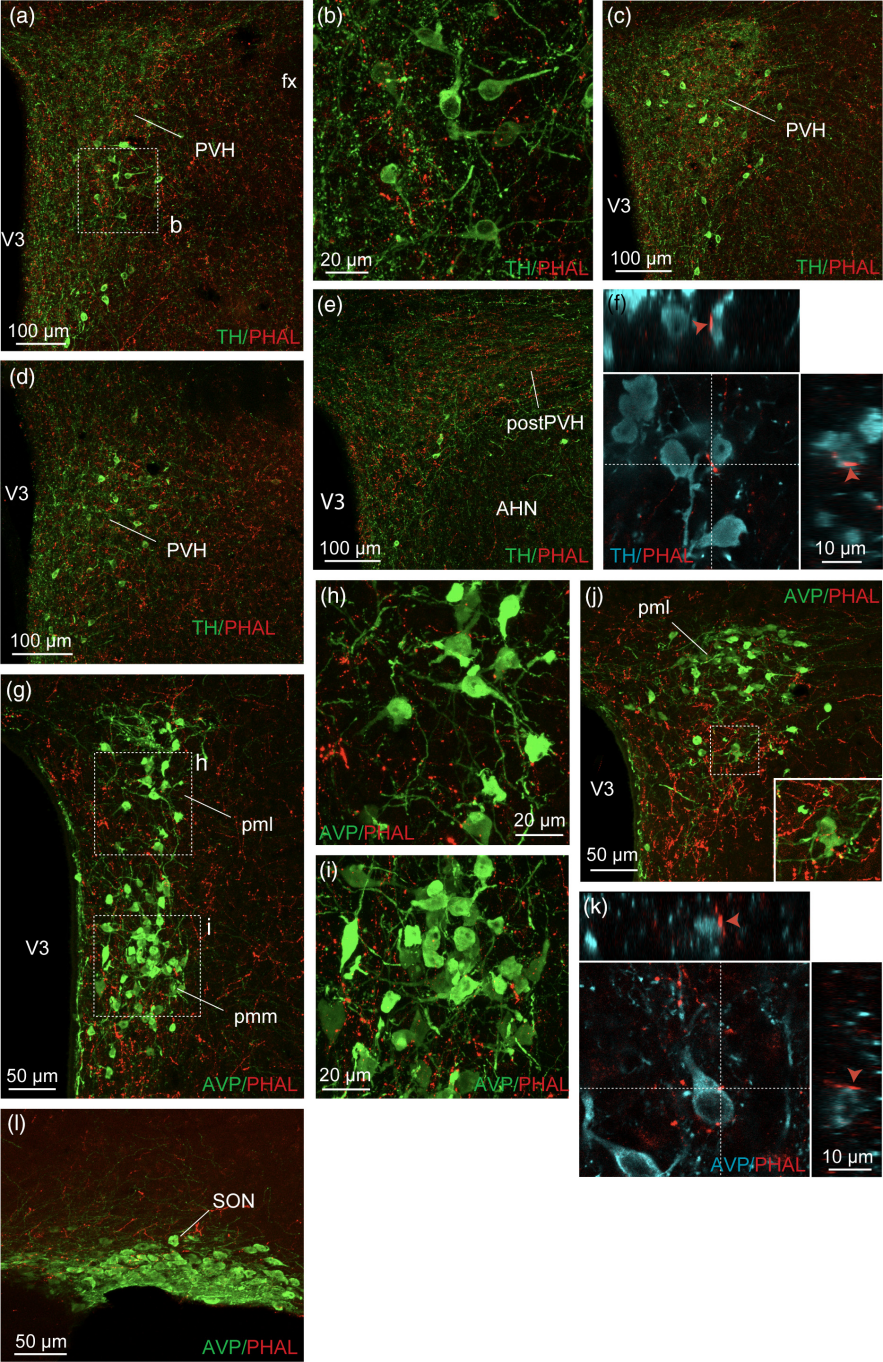
(a) Photomicrograph showing double immunodetection of PHAL‐positive fibers (red) from the dorsomedial BNST and catecholaminergic neurons labeled with tyrosine hydroxylase (TH) (green) in the paraventricular nucleus of the hypothalamus (PVH) (experiment BNST#2). (b) Image of the inset shown in (a) and illustrating PHAL‐positive fibers (red) from the dorsomedial BNST in the vicinity of TH‐positive neurons (green, experiment BNST#2). Photomicrograph showing double immunodetection of PHAL‐positive fibers (red) and catecholaminergic neurons labeled with tyrosine hydroxylase (TH) (green) in the caudal PVH (c, d) and in the posterior PVH (postPVH) (e). (f) Microphotograph illustrating the double immunodetection of PHAL‐positive axons (red) in contact with TH‐expressing neurons (cyan, experiment BNST#2). Inset on top in f shows the orthogonal view on the vertical line and inset on the right shows the orthogonal view of the horizontal line. Red arrows indicate the contacting site between PHAL‐positive terminals and TH neuron. (g) Photomicrograph showing PHAL‐positive fibers (red) from the dorsomedial BNST and arginine‐vasopressin (AVP) magnocellular labeled‐neurons (green) in the neuroendocrine PVH (experiment BNST#6). (h, i) High magnifications of the insets shown in (g) of PHAL‐labeled axons (red) from the dorsomedial BNST and AVP‐positive neurons (green) in the neuroendocrine PVH (experiment BNST#6). (j) Immunofluorescence of PHAL‐labeled axons (red) from the dorsomedial BNST and AVP‐positive neurons in the caudal neuroendocrine PVH (experiment BNST#6). The inset highlights a high magnification of PHAL‐positive axons in contact with AVP‐positive neurons. (k) Microphotographs illustrating the double immunodetection of PHAL‐positive axons (red) in contact with AVP‐expressing neurons (cyan, experiment BNST#6). Inset on top shows the orthogonal view on the vertical line and inset on the right shows the orthogonal view of the horizontal line. Red arrows indicate the contacting site between PHAL‐positive terminals and AVP neurons. (l) Immunofluorescence of PHAL‐labeled axons (red) from the dorsomedial BNST and AVP‐positive neurons in the SON (experiment BNST#6). AHN, anterior nucleus of the hypothalamus; fx, fornix; pml, posterior magnocellular part, lateral zone of the PVH; pmm, posterior magnocellular part, medial zone of the PVH; pv, periventricular nucleus; SON, the supraoptic nucleus; VMH, ventromedial nucleus of the hypothalamus; V3, third ventricle. Scale bars are shown in the figure [Color figure can be viewed at wileyonlinelibrary.com]

In several levels of the rostral neuroendocrine part of the PVH, many PHAL‐positive fibers were observed among parvicellular TH‐positive neurons (Hornby & Piekut, 1987) (Figure 5a–e). Putative synaptic boutons and varicosities were seen in close apposition of these catecholaminergic neurons (Figure 5b,f). In contrast, AVP‐containing areas mostly contained light dorsomedial BNST PHAL‐terminals (Figure 5g–j). Despite this weak axon density, we observed PHAL‐positive terminals in contact with AVP‐expressing neurons (Figure 5k). The supraoptic nucleus which also contains AVP‐positive magnocellular neurons, received only a sparse input from the dorsomedial BNST (Figure 5l).

The posterior part of the PVH can be divided into a medial parvicellular neuroendocrine part and an autonomic part that is more lateral (Figure 4i–k) (Bouyer & Simerly, 2013). The former contained a high PHAL‐positive axon density with terminal boutons seen in contact with neurons labeled with NeuN (Figure 4e,f). The latter displayed scattered PHAL‐positive fibers with a few axons running toward the lateral extremity of the nucleus (Figure 4i–k).

### 3.4 The dorsomedial, the capsule of the ventromedial, the tuberal nuclei and the lateral hypothalamic area

**FIGURE 6:**
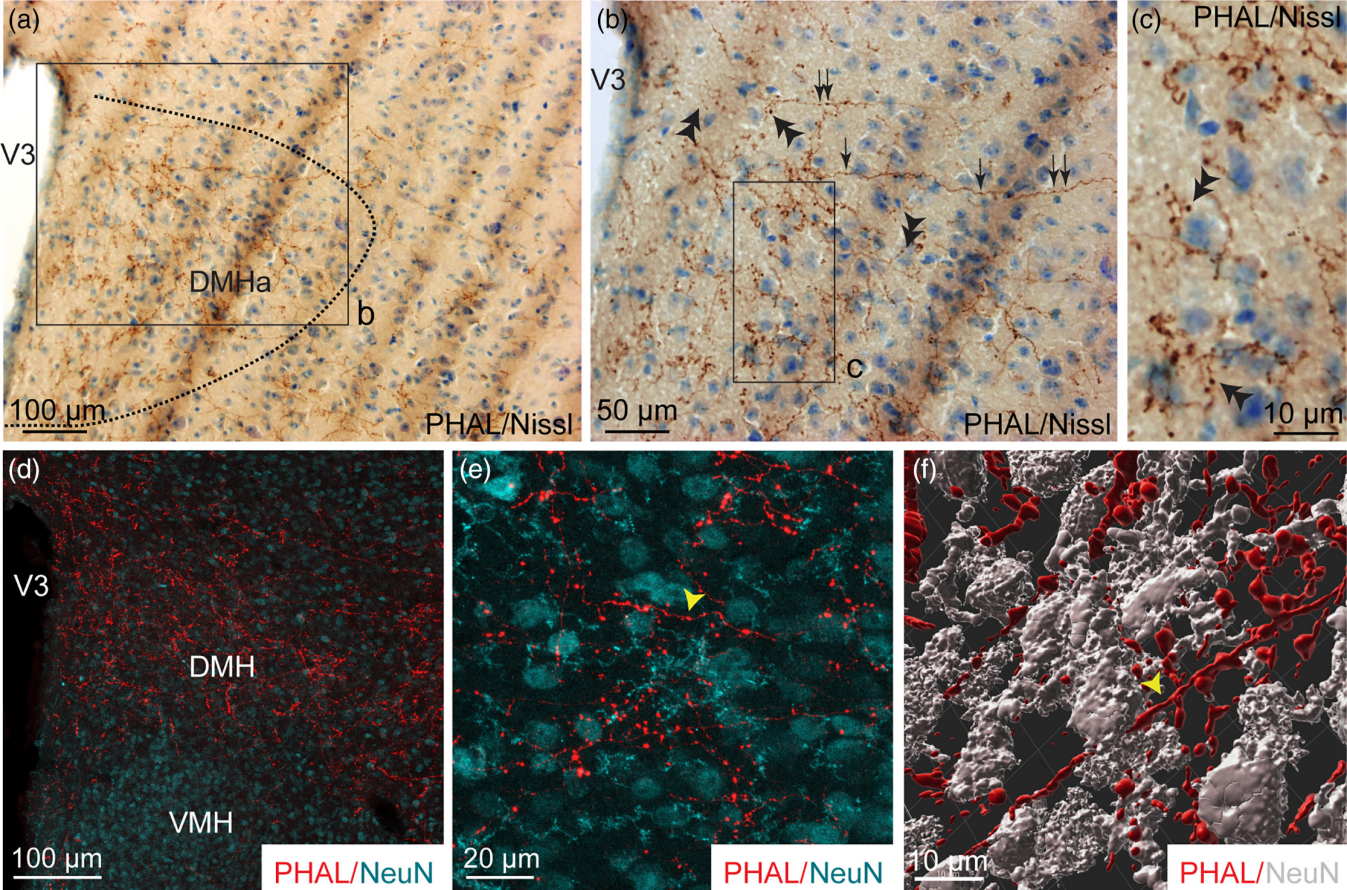
(a, b) Photomicrographs showing Nissl‐stained sections for cytoarchitectonic purposes of the DMH combined with enzymatic detection of PHAL from the dorsomedial BNST (experiment BNST#5). (c) High magnification of the inset shown in b. Arrows represent varicosities, double arrowheads show short collaterals with terminal boutons. (d) Microphotograph illustrating the double immunodetection of PHAL‐labeled fibers (red) from the dorsomedial BNST and NeuN‐positive neurons (cyan) in the DMH. The VMH is devoid of PHAL fibers (experiment BNST#2). (e) High magnification of the double immunodetection of PHAL‐labeled fibers (red) and NeuN‐positive neurons (cyan) in the DMH. (f) 3D reconstruction using Imaris software of a PHAL‐positive axon (red, yellow arrowhead) from the dorsomedial BNST displaying several short collaterals in contact with neurons (grey) labeled with NeuN in the DMH. Scale bars are shown in the figure [Color figure can be viewed at wileyonlinelibrary.com]

PHAL‐labeled fibers from the dorsomedial division of the BNST provided moderate to intense innervation to areas surrounding the fornix such as the DMH, the dorsal part of the capsule of the VMH, the tuberal nucleus and the LHA, ignoring the border limits between these territories (Figures 2d–f, 3c–d, and 6a,d). Each of these areas is nonetheless characterized by a singular genetic signature, with neurons expressing MCH and TH in the DMH, MCH in the capsule of the VMH and MCH and ORX in the LHA (Bittencourt, 2011; Croizier et al., 2010; Cvetkovic et al., 2004; Lein et al., 2007).

**FIGURE 7:**
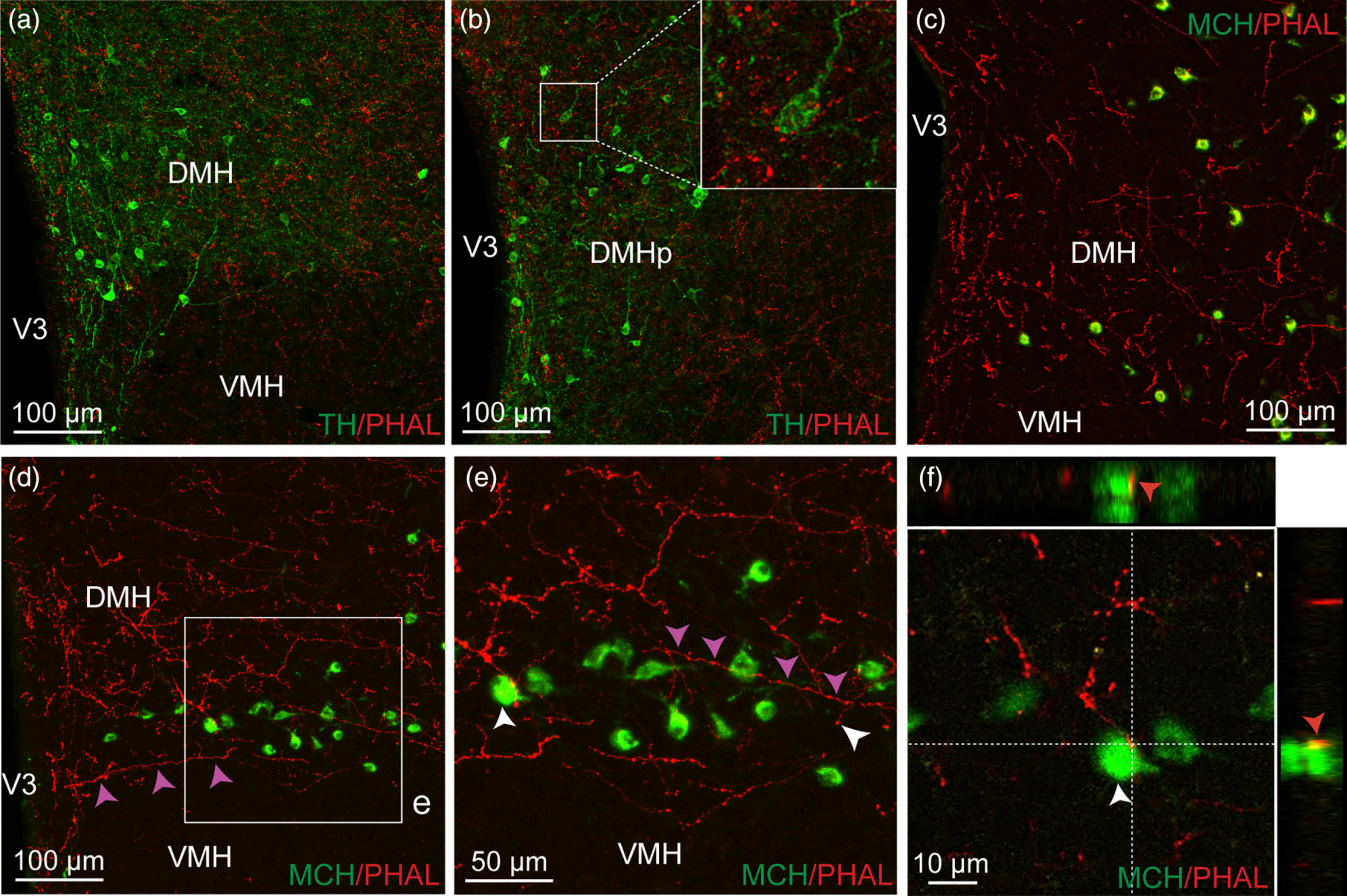
Immunofluorescence of PHAL‐labeled axons (red) from the dorsomedial BNST and tyrosine hydroxylase (TH)‐positive neurons (green) in the rostral (a) and posterior DMH (b). Inset in b shows higher magnification of PHAL‐positive axons surrounding TH‐positive neurons (experiment BNST#2). Microphotographs illustrating the double immunolabeling of PHAL‐positive fibers (red) from the dorsomedial BNST with MCH‐labeled neurons (green) in the DMH (c, experiment BNST#5) and in the capsule of the VMH (d, e, experiment BNST#6). One running axon (red) is highlighted by pink arrows and display varicosities. A PHAL‐positive axon (red) is in contact with one melanin‐concentrating hormone (MCH)‐labeled neuron (green) in the capsule of the VMH (f). Inset on top in f shows the orthogonal view on the vertical line and inset on the right shows the orthogonal view of the horizontal line. Red arrows indicate the contacting site between PHAL‐positive terminals and MCH neuron. DMH, dorsomedial nucleus of the hypothalamus; VMH, ventromedial nucleus of the hypothalamus; V3, third ventricle. Scale bars are shown in the figure [Color figure can be viewed at wileyonlinelibrary.com]

A moderate number of PHAL‐labeled axons was found throughout the DMH (Figures 2e,f, 3c, 6a,d, and 7a–c). Some of them extended from the adjacent lateral area and displayed abundant varicosities, collaterals, boutons‐of‐passage and terminal boutons (Figures 3c and 6a–c). The Nissl counterstaining evocated axosomatic contacts (Figure 6b,c) which was confirmed after double labeling for PHAL/NeuN and 3D reconstruction (Figure 6e,f). We observed PHAL fibers in close apposition of TH‐immunolabeled neurons in the posterior DMH (Figure 7a,b). However, we were not able to observe PHAL axons in contact with the few MCH cell bodies that were observed within the borders of the DMH (Figure 7c).

A few such contacts by axons‐of‐passage coming from lateral hypothalamic areas were observed on MCH neurons localized in the dorsal part of the capsule of the VMH (Figure 7d–f).

**FIGURE 8:**
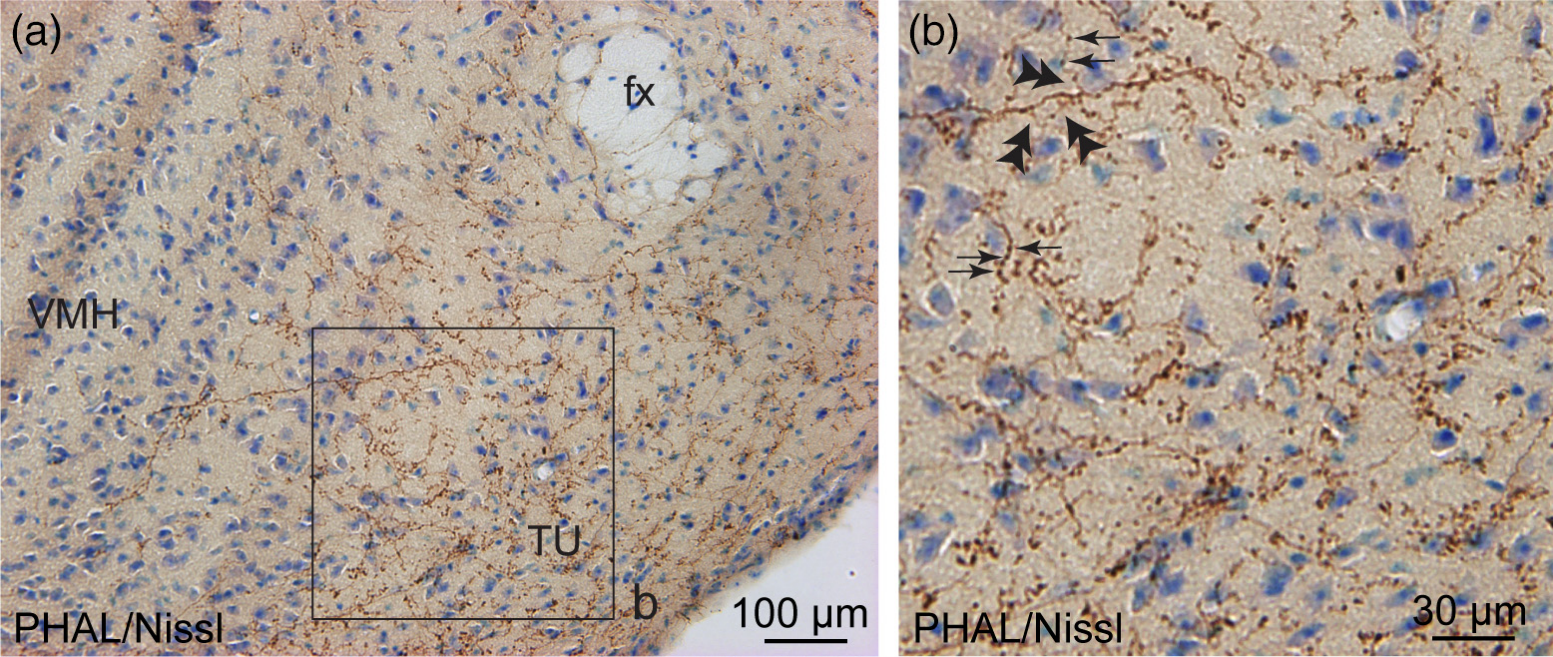
(a) Photomicrograph showing Nissl‐stained sections for cytoarchitectonic purposes of the tuberal nucleus (TU) combined with enzymatic detection of PHAL from the dorsomedial BNST (experiment BNST#6). (b) High magnification of the inset shown in a. Arrows represent varicosities along a PHAL‐positive axon, double arrowheads show short collaterals with terminal boutons. fx, fornix; VMH, ventromedial nucleus of the hypothalamus. Scale bars are shown in the figure [Color figure can be viewed at wileyonlinelibrary.com]

The core of the VMH was devoid of PHAL‐positive axons (Figures 2d–f and 3c,d). However, the tuberal nucleus adjacent to the ventrolateral part of the VMH received moderate to intense dorsomedial BNST projections (Figures 2d–f, 3d, and 8a). Numerous varicosities, short collaterals ended by putative terminal boutons were observed in the immediate vicinity of Nissl‐stained perikarya (Figure 8b).

**FIGURE 9:**
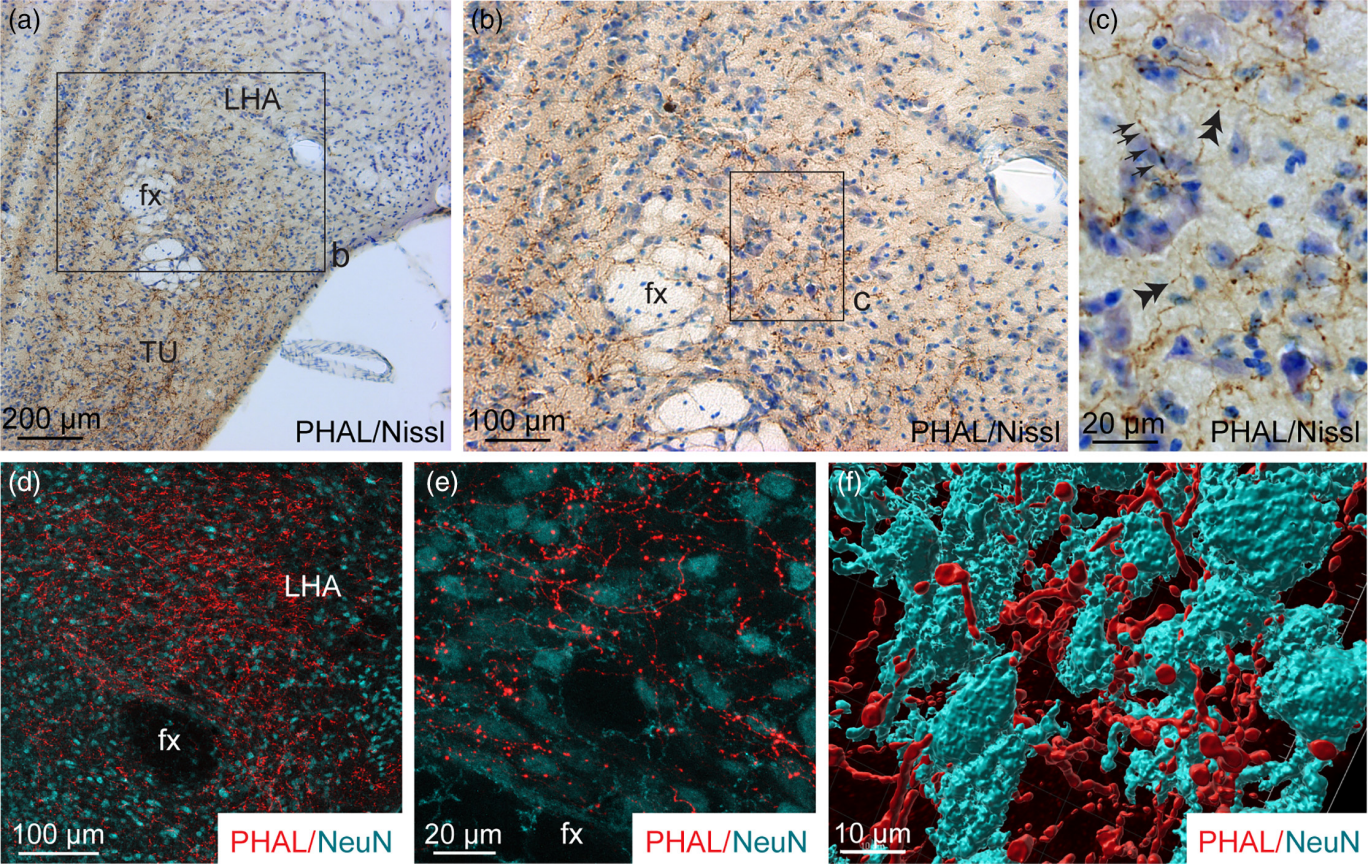
(a, b) Photomicrographs showing Nissl‐stained sections for cytoarchitectonic purposes of the LHA combined with enzymatic detection of PHAL from the dorsomedial BNST (experiment BNST#5). (c) High magnification of the inset shown in b. Arrows represent varicosities along a PHAL‐positive axon, double arrowheads show short collaterals with terminal boutons. (d) Microphotograph illustrating the double immunodetection of PHAL‐labeled fibers (red) from the dorsomedial BNST and NeuN‐positive neurons (cyan) in the LHA (experiment BNST#2). (e) High magnification of the double immunodetection of PHAL‐labeled fibers (red) and NeuN‐positive neurons (cyan) in the LHA. (f) 3D reconstruction using Imaris software of a PHAL‐positive axon (red) from the dorsomedial BNST in contact with neurons (cyan) labeled with NeuN in the LHA. fx, fornix; LHA, lateral hypothalamic area; TU, tuberal nucleus. Scale bars are shown in the figure [Color figure can be viewed at wileyonlinelibrary.com]

Finally, the LHA extends from the anterior hypothalamus in PVH‐containing sections (Bregma −0.70, Franklin and Paxinos Mouse Atlas, 2008) until the most posterior part of the tuberal hypothalamus (Bregma −2.54, Franklin and Paxinos Mouse Atlas, 2008). PHAL labeled axons were abundant mostly dorsal and dorsolateral to the fornix (Bregma −1.46 to −1.94, Franklin and Paxinos Mouse Atlas, 2008), where numerous MCH and ORX neurons are observed (Figures 2d–f and 9a,b,d). Dense plexus (branches and boutons) was found close to and potentially in contact with Nissl‐positive cells (Figures 2e,f and 9b,c) which was confirmed after NeuN‐immunolabeling and 3D reconstruction (Figure 9e,f). In contrast, PHAL‐labeled fibers observed in a very lateral position, close to the cerebral peduncle were cut in cross‐section indicating that they only follow the medial forebrain bundle toward the caudal brainstem (Figures 2f and 3d).

**FIGURE 10:**
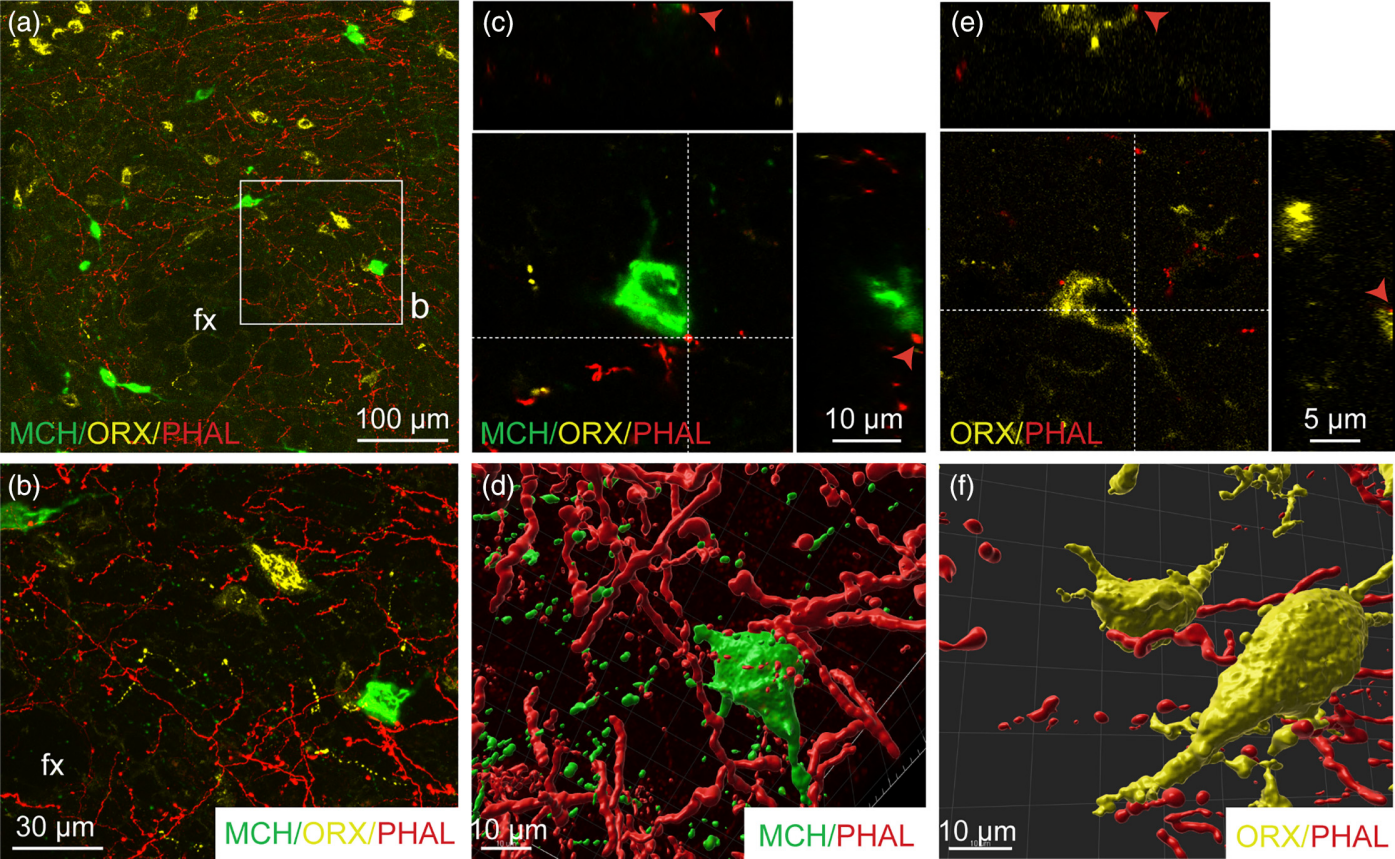
(a) Triple immunofluorescence of PHAL‐labeled axons (red) from the dorsomedial BNST, melanin‐concentrating hormone (MCH, green)‐ and orexin (ORX, yellow)‐positive neurons in the perifornical area and the LHA (experiment BNST#6). (b) High magnification of the inset shown in a. A PHAL‐positive axon (red) is in contact with one MCH‐labeled neuron (c, green) and with one ORX neurons (d, yellow) in the perifornical area. Insets on top in c and d show the orthogonal view on the vertical line and insetson the right show the orthogonal view of the horizontal line. Red arrows indicate the contacting site between PHAL‐positive terminals and MCH or ORX neurons. 3D reconstruction using Imaris software of PHAL‐positive axons (red) from the dorsomedial BNST in contact with MCH (green) and ORX (yellow) neurons in the perifornical area. Fx, fornix. Scale bars are shown in the figure [Color figure can be viewed at wileyonlinelibrary.com]

MCH and ORX neurons are the two main neuron populations of the tuberal LHA (Bittencourt et al., 1992; Croizier et al., 2010; Hahn & Swanson, 2010; Peyron et al., 1998; Watts & Sanchez‐Watts, 2007). We revealed abundant contacts of PHAL‐positive terminals with MCH and ORX immunolabeled‐neurons in the LHA and in the perifornical area (Figure 10a) notably after 3D reconstruction (Figure 10b–f).

**FIGURE 11:**
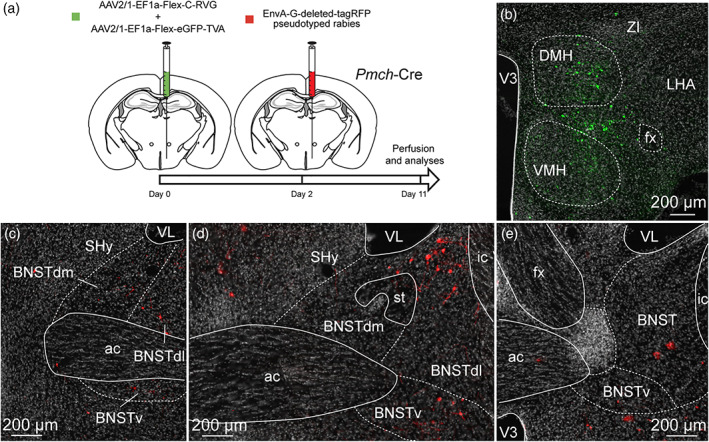
(a) Experimental approach. A mix of AAV‐EF1a‐Flex‐C‐RVG and AAV‐EF1a‐Flex‐eGFP‐TVA was injected at day 0 in the DMH and the dorsal part of the capsule of the VMH of 9‐ to 10‐week‐old *Pmch*‐Cre male mice. Two days later, mice received injection of EnvA‐G‐deleted‐tagRFP pseudotyped rabies. Animals were perfused nine days later for further analyses. (b) Photomicrograph of the site of stereotactic injection of the viruses. eGFP‐positive cells (green) were infected with the AAV‐EF1a‐Flex‐eGFP‐TVA. (c–e) Microphotographs illustrating the distribution of neurons projecting onto MCH neurons of the DMH and the capsule of the VMH (tRFP, red), at several levels of the anterior bed nucleus of the stria terminalis (BNST). tRFP‐positive cells are observed in the dorsomedial and lateral division of the BNST as wells as in the ventral division. ac, anterior commissure; BNSTdm, dorsomedial division of the BNST; BNSTdl, dorsolateral division of the BNST; BNSTv, ventral division of the BNST; DMH, dorsomedial nucleus of the hypothalamus; fx, fornix; ic, internal capsule; LHA, lateral hypothalamic area; shy, septohypothalamic nucleus; st, stria terminalis; VMH, ventromedial nucleus of the hypothalamus; VL, lateral ventricle; V3, third ventricle; ZI, zona incerta. Scale bars are shown in the figure [Color figure can be viewed at wileyonlinelibrary.com]

**FIGURE 12:**
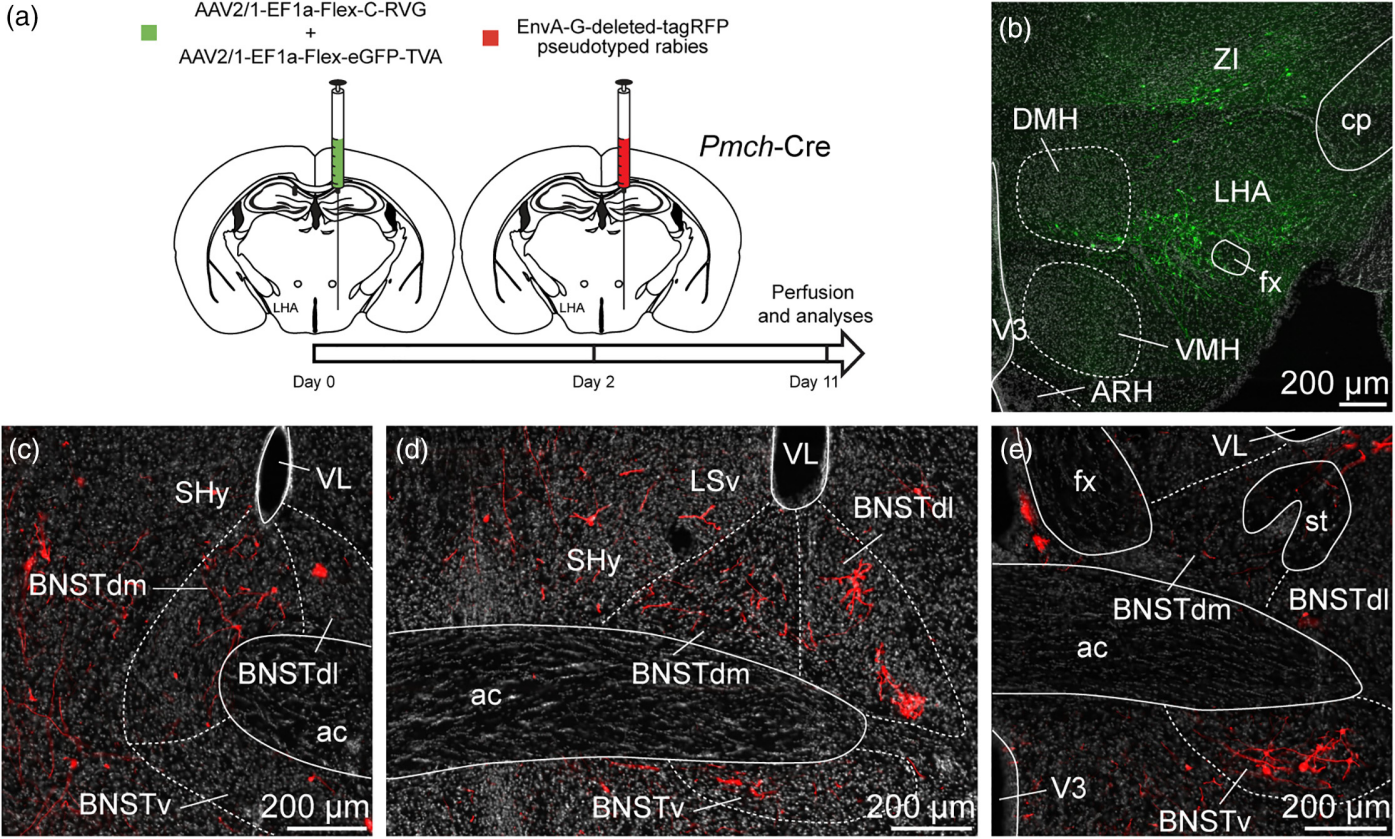
(a) Experimental approach. A mix of AAV‐EF1a‐Flex‐C‐RVG and AAV‐EF1a‐Flex‐eGFP‐TVA was injected at day 0 in the perifornical area and the LHA of 9‐ to 10‐week‐old *Pmch*‐Cre male mice. Two days later, mice received injection of EnvA‐G‐deleted‐tagRFP pseudotyped rabies. Animals were perfused 9 days later for further analyses. (b) Photomicrograph of the site of stereotactic injection of the viruses. eGFP‐positive cells (green) were infected with the AAV‐EF1a‐Flex‐eGFP‐TVA. (c–e) Microphotographs illustrating the distribution of neurons projecting onto MCH neurons of the perifornical area and the LHA (tRFP, red), at several levels of the anterior bed nucleus of the stria terminalis (BNST). tRFP‐positive cells are observed in the dorsomedial and lateral division of the BNST as wells as in the ventral division. ac, anterior commissure; ARH, arcuate nucleus of the hypothalamus; BNSTdm, dorsomedial division of the BNST; BNSTdl, dorsolateral division of the BNST; BNSTv, ventral division of the BNST; cp, cerebral peduncle; DMH, dorsomedial nucleus of the hypothalamus; fx, fornix; LHA, lateral hypothalamic area; LSv, ventral part of the lateral septum; MCH, melanin‐concentrating hormone; shy, septohypothalamic nucleus; st, stria terminalis; VMH, ventromedial nucleus of the hypothalamus; VL, lateral ventricle; V3, third ventricle; ZI, zona incerta. Scale bars are shown in the figure [Color figure can be viewed at wileyonlinelibrary.com]

**FIGURE 13:**
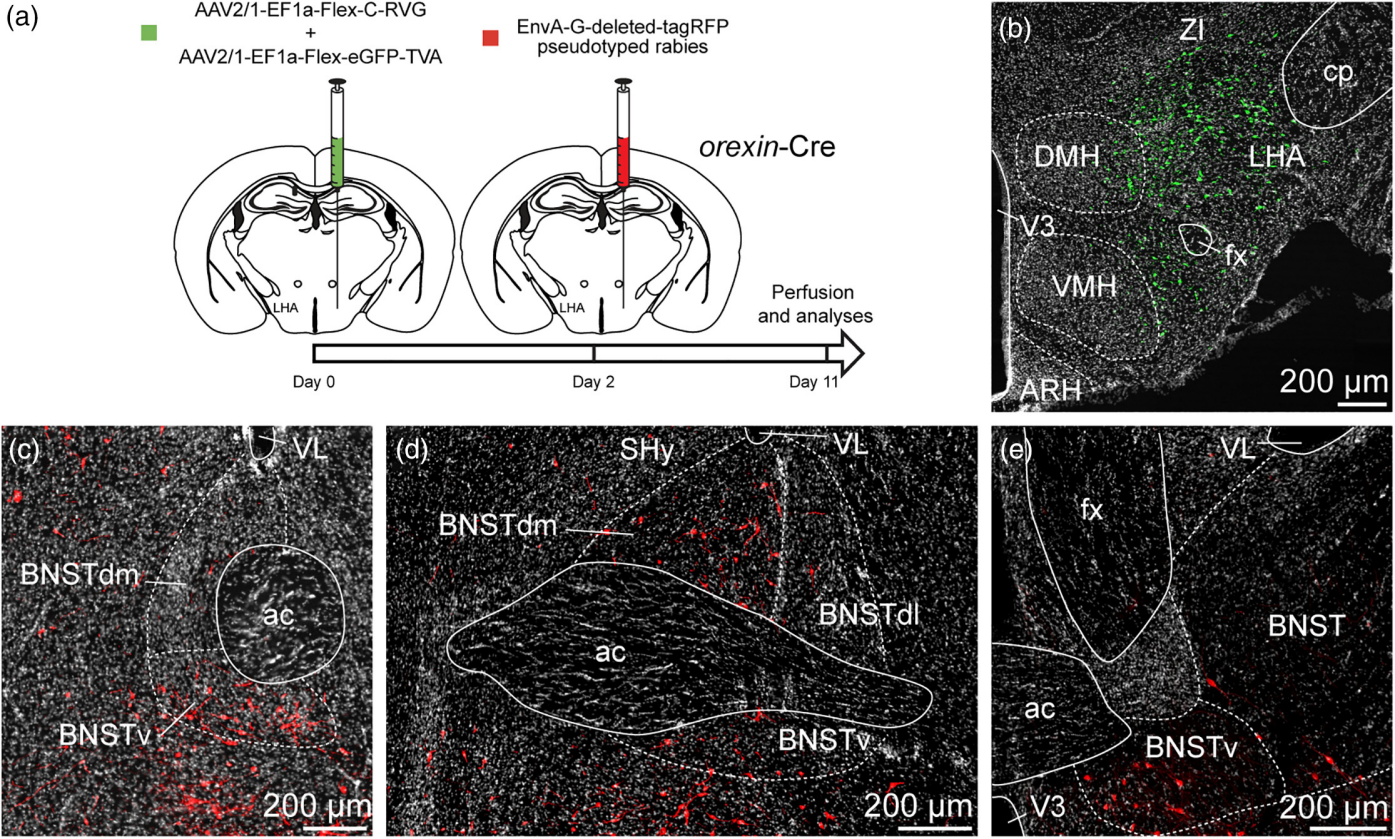
(a) Experimental approach. A mix of AAV‐EF1a‐Flex‐C‐RVG and AAV‐EF1a‐Flex‐eGFP‐TVA was injected at day 0 in the perifornical area and the LHA of 12 weeks old *orexin*‐Cre male mice. Two days later, mice received injection of EnvA‐G‐deleted‐tagRFP pseudotyped rabies. Animals were perfused 9 days later for further analyses. (b) Photomicrograph of the site of stereotactic injection of the viruses. eGFP‐positive cells (green) were infected with the AAV‐EF1a‐Flex‐eGFP‐TVA. (c–e) Microphotographs illustrating the distribution of neurons projecting onto orexin (ORX) neurons of the perifornical area and the LHA (tRFP, red), at several levels of the anterior bed nucleus of the stria terminalis (BNST). tRFP‐positive cells are observed in the dorsomedial and lateral division of the BNST as wells as in the ventral division. ac, anterior commissure; ARH, arcuate nucleus of the hypothalamus; BNSTdm, dorsomedial division of the BNST; BNSTdl, dorsolateral division of the BNST; BNSTv, ventral division of the BNST; cp, cerebral peduncle; DMH, dorsomedial nucleus of the hypothalamus; fx, fornix; LHA, lateral hypothalamic area; LSv, ventral part of the lateral septum; shy, septohypothalamic nucleus; st, stria terminalis; VMH, ventromedial nucleus of the hypothalamus; VL, lateral ventricle; V3, third ventricle; ZI, zona incerta. Scale bars are shown in the figure [Color figure can be viewed at wileyonlinelibrary.com]

To ensure the presence of synapses between the neurons of the anterodorsal divisions of the BNST and MCH neurons of the capsule of the VMH, the DMH and the LHA, we took advantage of the retrograde monosynaptic tracing. For this, we used a Cre‐dependent helper adeno‐associated viruses (AAV) expressing TVA receptor for the avian sarcoma leucosis virus glycoprotein (EnvA; AAV2/1‐EF1a‐Flex‐eGFP‐TVA) and RG (rabies envelope glycoprotein; AAV2/1‐EF1a‐Flex‐C‐RVG) associated with an EnvA‐G‐deleted‐tagRFP pseudotyped rabies virus (Figure 11a) (González et al., 2016; Vélez‐Fort et al., 2014). When used with *Pmch*‐Cre mice, TVA and RG allow the rabies infection in MCH neurons of the capsule of the VMH, the DMH and the LHA as shown by GFP fluorescence (Figures 11b, 12a,b, and 13a,b). After 9 days, we examined the RFP‐positive signal in several subdivisions of the anterior BNST. When injected in the capsule of the VMH and the DMH, RFP‐positive cells were mostly detected in the dorsolateral parts, as well as in the ventral parts of the anterior BNST and to a lesser extend in the dorsomedial division (Figure 11c–e). After injection in the perifornical LHA, monosynaptic inputs to MCH neurons were detected from both the dorsomedial and dorsolateral parts, as well as in the ventral parts of the anterior BNST (Figure 12c–e). Monosynaptic inputs to ORX neurons of the LHA were observed in the same BNST divisions as described for MCH (Figure 13c–e).

### 3.5 The arcuate nucleus

**FIGURE 14:**
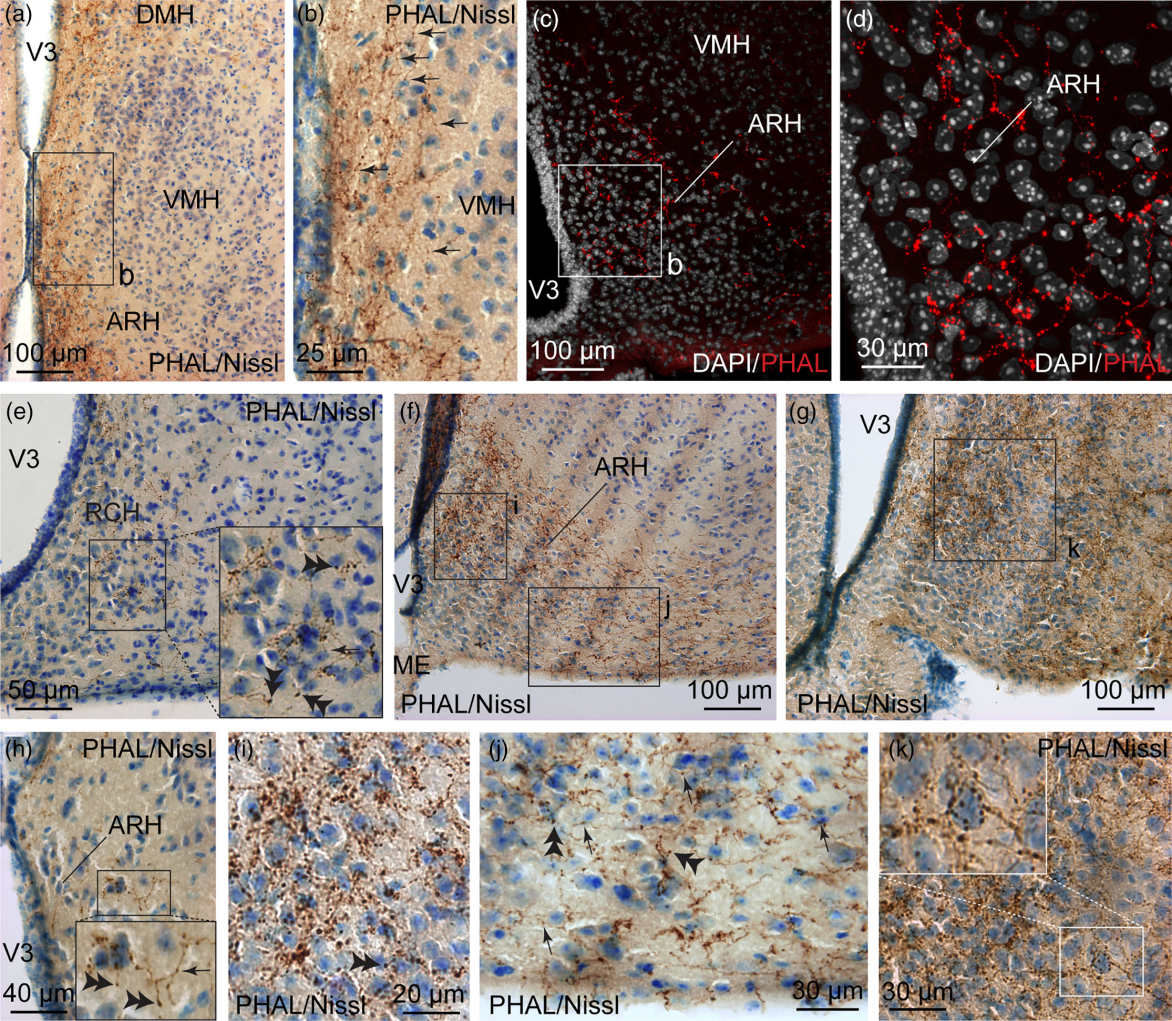
(a) Photomicrograph showing Nissl‐stained sections for cytoarchitectonic purposes combined with enzymatic detection of PHAL from the dorsomedial BNST (experiment BNST#6). Running PHAL axons are observed along the third ventricle to reach the dorsal part of the ARH. (b) High magnification of the inset shown in a. Arrows represent varicosities along PHAL‐positive axons. (c) Microphotograph showing PHAL‐immunolabeled fibers (red) from the dorsomedial division of the BNST and DAPI counterstained nuclei (white) in the ARH. (d) High magnification of the inset shown in c. PHAL‐labeled fibers are more abundant in the dorsomedial part of the ARH when compared to the ventrolateral area. Photomicrographs illustrating Nissl‐stained sections for cytoarchitectonic purposes combined with enzymatic detection of PHAL from the dorsomedial BNST in the RCH (e, experiment BNST#3), and in rostral (f) and caudal (g) levels of the ARH (experiment BNST#6). (h) High magnification of Nissl‐stained sections combined with enzymatic detection of PHAL from the dorsomedial BNST in the dorsal part of the caudal ARH. Inset in h shows varicosities (arrow) and short collaterals ended by terminal Bouton (double arrowheads). High magnifications of the of Nissl‐stained sections combined with enzymatic detection of PHAL from the dorsomedial BNST of the insets shown in f in the dorsomedial part of the ARH (i) and in the ventrolateral part of the ARH (j). Abundant varicosities (arrows) and terminal boutons are observed in the vicinity of nucleated cells (Nissl), especially in the dorsomedial part of the ARH. (k) High magnification of the of Nissl‐stained sections combined with enzymatic detection of PHAL from the dorsomedial BNST of the insets shown in g in the dorsomedial part of the posterior ARH. Inset in k shows high magnification of abundant terminal boutons contacting a nucleated cell (Nissl) in the dorsal part of the ARH. ARH, arcuate nucleus of the hypothalamus; DMH, dorsomedial nucleus of the hypothalamus; ME, medial eminence; RCH, retrochiasmatic area, VMH, ventromedial nucleus of the hypothalamus; V3, third ventricle. Scale bars are shown in the figure [Color figure can be viewed at wileyonlinelibrary.com]

From the dorsomedial division of the BNST, fibers traveled caudally and ventrally near the third ventricle following a periventricular pathway to reach the ARH (Figures 2d,e and 14a,b). These parallel running axons displayed abundant varicosities (Figure 14a,b). Varicosities, collaterals and putative terminal boutons were also generated at several levels of the ARH (Figure 14c–g) such as the retrochiasmatic area (Figure 14e), and in the rostral and caudal ARH (Figure 14f–k). The intensity of PHAL‐labeled axons was higher in dorsolateral ARH (Figures 2d,e and 14c,d,f,g). Individual cells located in the dorsal ARH received overwhelming dense grape‐like terminal boutons and potential synaptic contacts (Figure 14i,k), suggesting a significant innervation of specific ARH cells. Many varicosities and terminal boutons ending short collaterals were found in the ventrolateral part of the ARH (Figure 14j).

**FIGURE 15:**
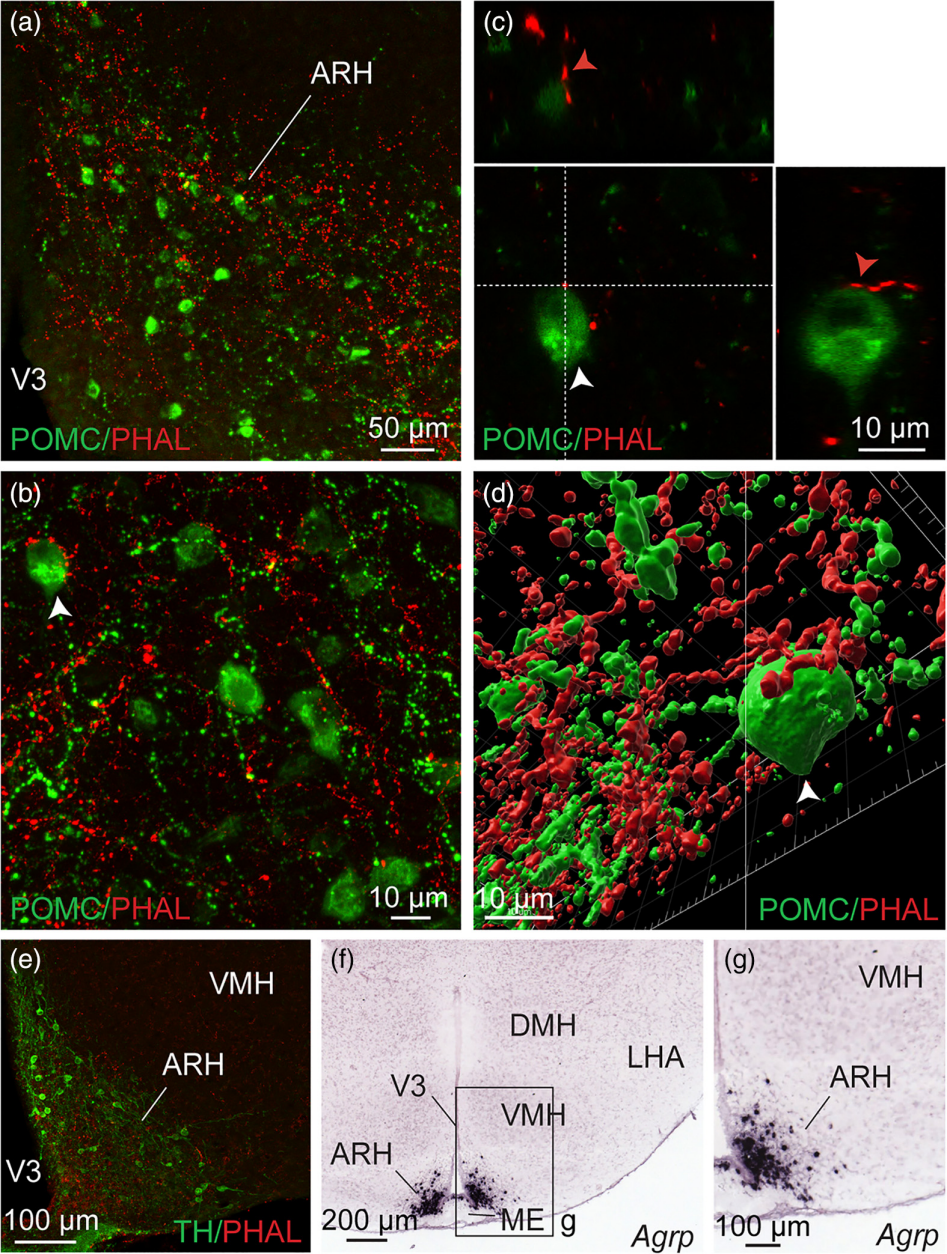
(a) Photomicrograph showing double immunodetection of PHAL‐positive fibers (red) from the dorsomedial BNST and proopiomelanocortin (POMC)‐labeled neurons (green) in the ARH (experiment BNST#6). (b) High magnification of the double immunodetection of PHAL‐positive fibers (red) from the dorsomedial BNST and POMC‐labeled neurons (green) in the ARH. Arrow highlights a POMC neuron contacted by PHAL‐positive axons. (c) Microphotographs illustrating the double immunodetection of PHAL‐positive axons (red) in contact with POMC‐expressing neurons (green) in the ARH (experiment BNST#2). Inset on top in shows the orthogonal view on the vertical line and inset on the right shows the orthogonal view of the horizontal line. Red arrows indicate the contacting site between PHAL‐positive terminals and POMC neuron. (d) 3D reconstruction using Imaris software of a PHAL‐positive axon (red) from the dorsomedial BNST in contact with POMC neurons (green) in the ARH. (e) Microphotograph illustrating double immunodetection of PHAL‐positive fibers (red) from the dorsomedial BNST and tyrosine hydroxylase (TH)‐labeled neurons (green) in the ARH (experiment BNST#2). (f) Image showing *Agouti‐related peptide* mRNA (*Agrp*)‐expressing neurons labeled by *in situ* hybridization in the ARH (image credit: Allen Institute, experiment #72283799). (g) High magnification of the inset shown in f. ARH, arcuate nucleus of the hypothalamus; DMH, dorsomedial nucleus of the hypothalamus; LHA, lateral hypothalamic area; ME, medial eminence; VMH, ventromedial nucleus of the hypothalamus; V3, third ventricle. Scale bars are shown in the figure [Color figure can be viewed at wileyonlinelibrary.com]

**FIGURE 16:**
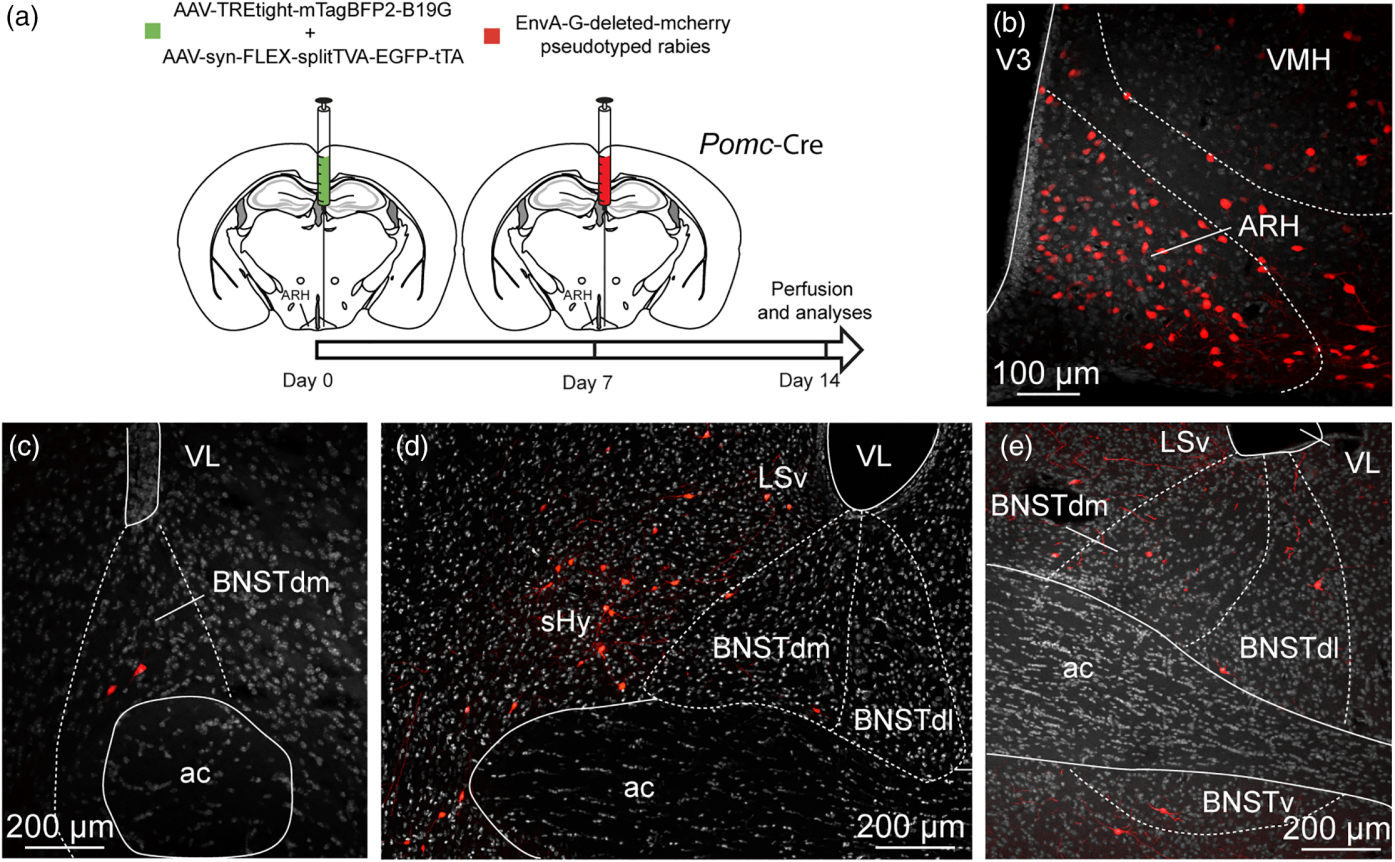
(a) Experimental approach. A mix of AAV‐TREtight‐mTagBFP2‐B19G and AAV‐syn‐FLEX‐splitTVA‐EGFP‐tTA was injected at day 0 in the ARH of 12 week‐old *Pomc*‐Cre male mice. Seven days later, mice received injection of EnvA‐G‐deleted‐mcherry pseudotyped rabies. (b) Photomicrograph of the site of stereotactic injection of the viruses. Mcherry‐positive cells (red) were infected with EnvA‐G‐deleted‐mcherry pseudotyped rabies. (c–e) Microphotographs illustrating the distribution of neurons projecting onto POMC neurons of the ARH (mcherry, red), at several levels of the anterior bed nucleus of the stria terminalis (BNST). Mcherry‐positive cells are observed in the dorsomedial and lateral division of the BNST as wells as in the ventral division. ac, anterior commissure; ARH, arcuate nucleus of the hypothalamus; BNSTdm, dorsomedial division of the BNST; BNSTdl, dorsolateral division of the BNST; BNSTv, ventral division of the BNST; LSv, ventral part of the lateral septum; shy, septohypothalamic nucleus; VMH, ventromedial nucleus of the hypothalamus; VL, lateral ventricle; V3, third ventricle. Scale bars are shown in the figure [Color figure can be viewed at wileyonlinelibrary.com]

**FIGURE 17:**
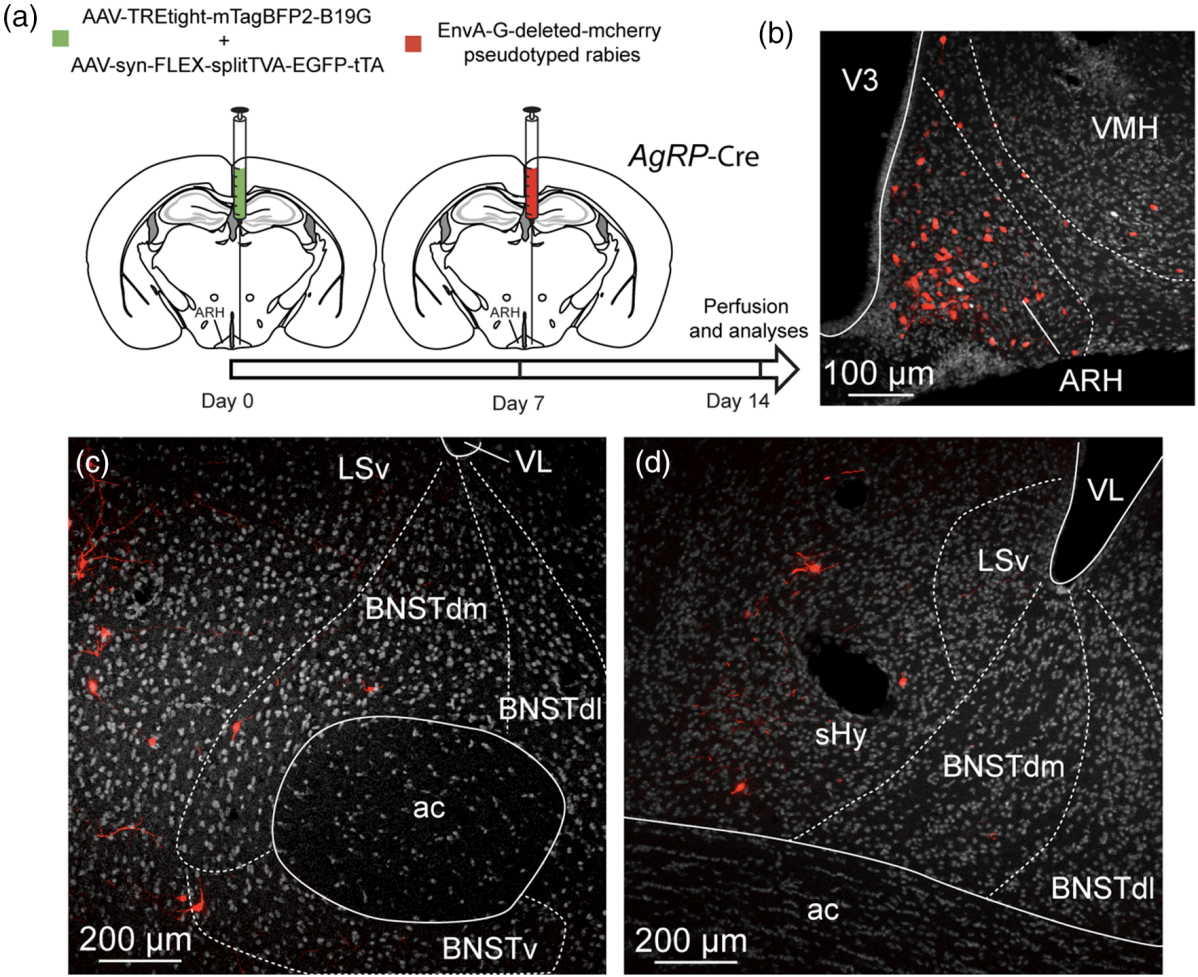
(a) Experimental approach. A mix of AAV‐TREtight‐mTagBFP2‐B19G and AAV‐syn‐FLEX‐splitTVA‐EGFP‐tTA was injected at day 0 in the ARH of 12 week‐old *AgRP*‐Cre male mice. Seven days later, mice received injection of EnvA‐G‐deleted‐mcherry pseudotyped rabies. (b) Photomicrograph of the site of stereotactic injection of the viruses. Mcherry‐positive cells (red) were infected with EnvA‐G‐deleted‐mcherry pseudotyped rabies. (c, d) microphotographs illustrating the distribution of neurons projecting onto AgRP neurons of the ARH (mcherry, red), at several levels of the anterior bed nucleus of the stria terminalis (BNST). Mcherry‐positive cells are observed in the dorsomedial division of the BNST as wells as in the ventral division. ac, anterior commissure; ARH, arcuate nucleus of the hypothalamus; BNSTdm, dorsomedial division of the BNST; BNSTdl, dorsolateral division of the BNST; BNSTv, ventral division of the BNST; LSv, ventral part of the lateral septum; shy, septohypothalamic nucleus; VMH, ventromedial nucleus of the hypothalamus; VL, lateral ventricle; V3, third ventricle. Scale bars are shown in the figure [Color figure can be viewed at wileyonlinelibrary.com]

The ARH contains neuronal populations that are topographically distributed (Croizier et al., 2010). Of which, POMC‐expressing neurons were mainly observed in the dorsolateral ARH while NPY/AgRP‐coexpressing neurons mostly localized in the ventromedial ARH close to the medial eminence and the third ventricle (Figure 15a,f,g) (Croizier et al., 2018; Lein et al., 2007). By using immunohistochemical approaches, confocal imaging and 3D reconstruction, we observed PHAL‐positive putative terminal boutons surrounding and in contact with POMC‐expressing neurons (Figure 15a–d). We also found PHAL terminals in contact with TH‐expressing neurons in the medial and lateral ARH (Figure 15e). Despite immunodetection of NPY‐ and AgRP‐positive fibers throughout the brain, we did not visualize positive staining in the arcuate cell bodies. However a more moderate number of PHAL‐positive axons were observed where NPY/AgRP neurons are found (Allen brain atlas, Figures 14c,f and 15f,g). To assess and confirm that neurons of the dorsomedial division of the BNST innervated both POMC and AgRP neurons, we used monosynaptic retrograde tracing by injecting these helper viruses: AAV‐TREtight‐mTagBFP2‐B19G and AAV‐syn‐FLEX‐splitTVA‐EGFP‐tTA), followed by the injection of EnvA‐G‐deleted‐mcherry pseudotyped rabies virus (Figures 16a and 17a) (Liu et al., 2017). When injected in the ARH of *Pomc*‐Cre animals (Figure 16b), monosynaptic inputs to POMC neurons (mcherry‐labeled) were mostly detected in the dorsomedial part and to a lesser extend in the dorsolateral, as well as in the ventral parts of the anterior BNST (Figure 16c–e). Interestingly, abundant mcherry‐positive cells were also observed in ventral part of the lateral septum and in the septohypothalamic nucleus (Figure 16d). When injected in the ARH of *Agrp*‐Cre mice (Figure 17b), monosynaptic spread led to detection of mcherry‐positive cells in the dorsomedial and ventromedial parts of the BNST (Figure 17c,d). We also noticed a few mcherry‐positive cells in the adjacent septohypothalamic nucleus (Figure 17d).

## 4 DISCUSSION

In the present study, we combined anterograde and retrograde tract tracing, histological and immunohistochemical approaches to describe the hypothalamic projections from the dorsomedial division of the BNST. The overall pattern of projections was similar to that described in rats by Dong and Swanson (2006), and we identified several hypothalamic nuclei including the PVH, the DMH, the tuberal nucleus, the LHA and the ARH as main targets of these projections. We provided a deeper insight on the innervation of specific neuronal populations within these structures including MCH, ORX, POMC and AgRP neurons.

### 4.1 The paraventricular nucleus

Our study showed a light to moderate innervation of distinct parts of the PVH from the dorsomedial BNST, in line with that described in rats (Dong & Swanson, 2006). Past retrograde studies confirmed the innervation of PVH neurons by neurons of the dorsomedial BNST and most of them have been performed in rats after fluorogold, true blue or cholera toxin B injection centered in the PVH (Cullinan et al., 1993; Moga & Saper, 1994; Prewitt & Herman, 1998; Sawchenko & Swanson, 1983; Spencer et al., 2005; Ulrich‐Lai et al., 2011). In mice, rabies‐based retrograde tracing showed a specific innervation of Corticotropin‐releasing factor receptor 1 (CRFR1)‐expressing PVH cells by dorsomedial BNST neurons (Jiang et al., 2018).

In the mouse and contrary to the rat, cytoarchitectonic limits between magnocellular and parvicellular subdivisions of the PVH are mostly undistinguishable (Biag et al., 2012; Simmons & Swanson, 2009). Nevertheless, the distributions of AVP‐ and CRH‐expressing neurons are segregated, somehow defining parvicellular and magnocellular compartments within the mouse PVH. We observed a denser innervation of the CRH‐containing area while the AVP‐expressing subdivisions contained few PHAL axons. Therefore, despite a poor compartmentalization of the PVH, our data are in agreement with the rat data, where parvicellular part is more densely innervated than magnocellular subdivision (Dong & Swanson, 2006; Simmons & Swanson, 2009). Although we were unable to verify that CRH neurons are innervated, we identified parvicellular TH‐positive cells as being targeted by these projections.

### 4.2 The dorsomedial nucleus

In mice, the DMH received a moderate innervation from the dorsomedial BNST also in line with that described in rats (Dong & Swanson, 2006). Retrograde tracing based on fluorogold injections in the DMH showed a concentration of neurons retrogradely labeled in the dorsomedial BNST of the rats (Thompson & Swanson, 1998). We observed PHAL‐positive fibers in the dorsomedial part of the DMH in contact with A11 TH‐positive dopaminergic neurons, but not of MCH cell bodies. The DMH is composed of a multitude of neurochemically distinct neurons potentially innervated by the dorsomedial BNST, including cholinergic neurons (Jeong et al., 2017), Fgf15‐expressing neurons (Picard et al., 2016), NPY neurons in the lateral parts (Bi et al., 2012; Lein et al., 2007). Moreover, the brain‐derived neurotrophic factor (BDNF) receptor, TrkB is expressed in all the divisions of the DMH (Liao et al., 2019). To our knowledge, no published data on the projections from the BNST to the above‐mentioned neurons are available in mice neither data on retrograde tracing from the aforementioned neurons. Our results although preliminary, seem to indicate that the dorsomedial BNST may innervate specifically some of those populations and not others (i.e. MCH).

### 4.3 The arcuate nucleus

As observed in rats (Dong & Swanson, 2006), very intense terminals are centered in the dorsomedial ARH in mice, where most of the POMC and dopamine neuroendocrine neurons are concentrated (Lein et al., 2007; Markakis & Swanson, 1997). We observed a much lighter input ventromedially and ventrolaterally where AgRP/NPY and growth hormone‐releasing hormone are, respectively, expressed (Lein et al., 2007; Sawchenko et al., 1985). In our study, the contact between terminal boutons with POMC neurons suggested the presence of synapses. In line with this observation, a previous study used retrograde tracing approach to show a specific innervation of POMC and AgRP neurons by the BNST (Wang et al., 2015), but the precise divisions of the BNST that were concerned were not clearly detailed. Here, we clarified that mostly dorsomedial divisions of the BNST projected onto POMC and AgRP neurons as well as the adjacent septohypothalamic nucleus. In agreement, one recent study confirmed the specific innervation of AgRP/NPY neurons by nociceptin‐expressing neurons of the dorsal divisions of the BNST (Smith et al., 2019).

### 4.4 The dorsal part of the capsule of the VMH and the tuberal nucleus

Similarly to that described in rats (Dong & Swanson, 2006), the core of the VMH is devoid of PHAL‐positive fibers arising from the dorsomedial subdivision of the BNST in mice. However, neurons of the capsule of the VMH are innervated and in particular MCH neurons. Other neuronal populations are also found in this restricted area such as the RF (Arg‐Phe) amide‐related peptides (RFRP) neurons (Legagneux et al., 2009).

The tuberal nucleus is immediately adjacent to the medial border of the VMH and interrupt the capsule of the VMH (Canteras et al., 1994). The tuberal nucleus provides intense innervation to nuclei containing neurosecretory motoneurons such as the parvicellular part of the PVH, the ARH and to the periaqueductal gray (Canteras et al., 1994). Taken with anatomical data, these observations suggest a role of the tuberal nucleus in the reproductive, defensive and more interestingly for this study in ingestive behaviors (Canteras et al., 1994).

### 4.5 The lateral hypothalamic area

The LHA is a complex and vast area receiving and sending projections from plethora of brain regions (Bittencourt et al., 1992; Hahn & Swanson, 2010, 2012, 2015). We decided to focus our study only in the region of the tuberal LHA where numerous MCH and ORX neurons are observed and where most of the projections from the dorsal divisions of the BNST end in rats. Our observation highlighted a strong innervation of the LHA and the perifornical area by the neurons of the dorsal and ventral divisions of the BNST in mice. In agreement with our data, the highest amount of retrograde labeling from what Hahn and Swanson (2010) name the LHAs (suprafornical region of the LHA, dorsal to the fornix, at the level of the DMH, VMH and ARH), is accumulated in the dorsomedial subdivision of the anterior BNST in rats. This subdivision of the LHA, in addition to more lateral areas, contain numerous MCH and ORX neurons. In line with published data, we described that neurons of the dorsomedial and lateral parts of the BNST innervate MCH and ORX neurons of the LHA. Indeed, cholecystokinin (CCK), and CRH‐expressing neurons of the dorsomedial and dorsolateral divisions of the BNST, respectively, target ORX (or Hypocretin) neurons, with 60% of them targeted by BNST^CRH^ neurons against 12.5% were contacted by BNST^CCK^ neurons, suggesting a topographic organization of the BNST projections onto the LHA and ORX neurons in particular. To note, BNST^CRH^ and BNST^CCK^ neurons remain mainly connected to non‐ORX neurons (Giardino et al., 2018).

### 4.6 Off‐target control injections of PHAL

One of our PHAL injections targeted both the dorsomedial subdivision of the BNST and a small part of the adjacent septohypothalamic nucleus (ventral part of the lateral septum). Previous studies performed in rats, described a strong innervation of the dorsomedial and ventrolateral parts of the ARH by the ventral part of the lateral septum (Risold & Swanson, 1997). In our study, we cannot exclude that terminal boutons observed in contact with ARH cells come from the ventral part of the lateral septum. However, we observed similar contacts of PHAL‐positive fibers with ARH cells after injection of PHAL exclusively restricted to the dorsomedial division of the BNST.

Two cases were injected in both the dorsomedial and dorsolateral parts of the BNST potentially including the oval nucleus. Data in rats showed an absence of innervation of the ARH by the oval nucleus of the BNST (Dong et al., 2001). However, in these two cases, we were able to visualize PHAL‐labeled fibers in the ARH, suggesting that these projections probably only arose from the dorsomedial division of the BNST. Nonetheless, the projections from the oval nucleus strongly innervate the LHA. In this study we cannot exclude that a significant proportion of PHAL‐labeled fibers observed in the LHA also come from the dorsolateral division of the BNST.

### 4.7 Monosynaptic retrograde tracing

Applying the monosynaptic retrograde tracing method has incredibly increased over the last few years and appeared as a new and efficient tool in the structural characterization of neurocircuits (González et al., 2016; Krashes et al., 2014; Liu et al., 2017; Wang et al., 2015). In 2015, Wang et al. took advantage of this approach to depict the distribution of monosynaptic inputs of both arcuate POMC and AgRP neurons throughout the brain. While covering areas from the forebrain to the brainstem, the list of divisions and specific nuclei containing presynaptic neurons has not been clearly itemized at least in the forebrain. Secondly, the distribution of presynaptic neurons suggested limitation in the spread of the strain of the rabies virus from the arcuate, limiting its accumulation in remote areas such as the forebrain. Indeed, the use of B19 rabies strain has been shown not to efficiently spread when compared to N2c strain (Reardon et al., 2016). In this study, we used B19 strain, putatively restricting its spread and potentially influencing the number of presynaptic neurons. However, it should not influence the distribution of retrogradely‐labeled neurons.

### 4.8 Functional considerations of the dorsomedial BNST projections to hypothalamic areas

**FIGURE 18:**
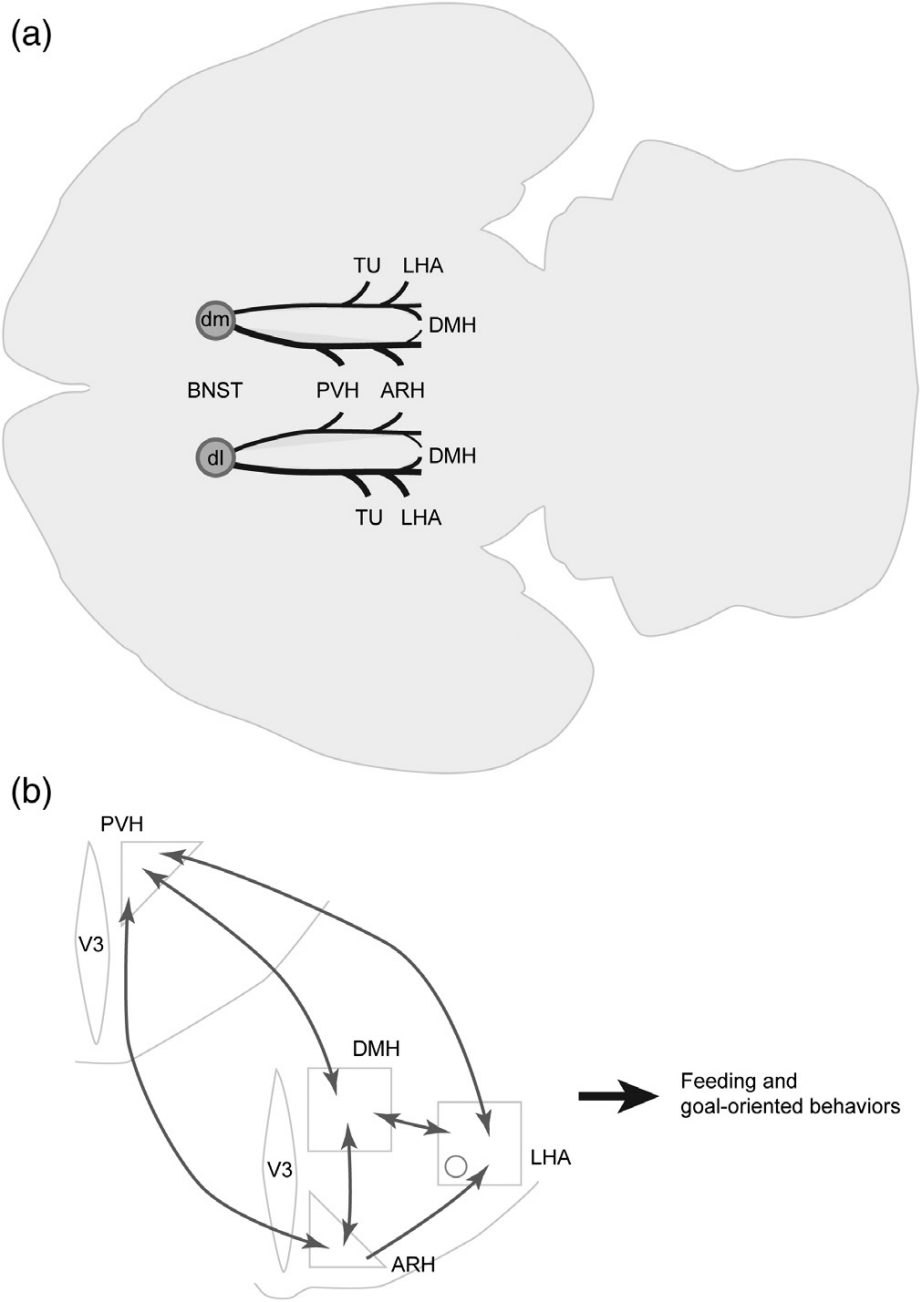
(a) Sagittal view of a brain showing general organization of the projections from the dorsomedial (BNSTdm) and dorsolateral (BNSTdl) divisions of the bed nucleus of the stria terminalis (BNST) to the studied hypothalamic nuclei. The relative strength of each pathway is proportional to the thickness of the black lines. Projections from the BNSTdm mostly innervate periventricular nuclei such as the paraventricular (PVH), the dorsomedial (DMH), the arcuate (ARH) nuclei of the hypothalamus, while those arising from BNSTdm innervate more lateral areas such as the lateral hypothalamic area (LHA). The flatmap is based on Franklin and Paxinos (2008). (b) Schematic illustrating the connections (dark grey) between the hypothalamic nuclei. The PVH, DMH, ARH and LHA are strongly interconnected. V3, third ventricle

Our study reveals that projections from the dorsomedial division of the BNST are connected to hypothalamic nuclei somehow linked to motivation and feeding behaviors such as the PVH (Atasoy et al., 2012; Betley et al., 2013; Hill, 2012), the DMH (Engström et al., 2020; Garfield et al., 2016; Jeong et al., 2017; Liao et al., 2019; Ryan et al., 2013), the LHA (Giardino et al., 2018; González et al., 2016; Jennings et al., 2013; Stuber & Wise, 2016) and the ARH (Timper & Brüning, 2017; van der Klaauw & Farooqi, 2015) (Figure 18).

Indeed, the dorsomedial division of the BSNT directly projects onto the DMH that is part of highly interconnected hypothalamic regions forming the visceromotor pattern generator network in rats (Thompson & Swanson, 2003). The DMH is composed of various neurons that are somehow involved in central feeding control and emotional component of eating behavior (Bello & Hajnal, 2010; Bi et al., 2012; de La Serre et al., 2016; Liao et al., 2019; Narayanan et al., 2010; Ryan et al., 2013; Volkow et al., 2003; Yang et al., 2009). These nuclei of the visceromotor pattern generator network innervate the autonomic system (Saper et al., 1976) and neuroendocrine motoneurons of the magnocellular (AVP, OXT) and parvicellular (CRH, TRH, SST, GRH, TH and GnRH) neurosecretory systems (Thompson & Swanson, 2003) known to control feeding behavior (Atasoy et al., 2012; Betley et al., 2013; Krashes et al., 2014; Lawson, 2017).

In agreement with published data, our study revealed in the tuberal hypothalamus, a strong innervation of MCH and ORX neurons of the LHA and POMC and AgRP neurons of the ARH, all well‐known effectors in the control of appetitive, aversive and goal‐oriented behaviors (Diniz & Bittencourt, 2017; Giardino et al., 2018; González et al., 2016; Jennings et al., 2013; Smith et al., 2019; Sohn, 2015; Stuber & Wise, 2016). Interestingly, in addition of being directly innervated by neurons of the dorsomedial divisions of the BNST, the aforementioned hypothalamic nuclei are also strongly interconnected suggesting a reinforcement of their role in feeding and motivated behaviors (Figure 18). In particular, PVH and DMH neurons are interconnected (Thompson et al., 1996; Thompson & Swanson, 1998) and both innervate arcuate neurons such as POMC and AgRP to control for instance feeding (Garfield et al., 2016; Jeong et al., 2017; Krashes et al., 2014; Wang et al., 2015). In return, ARH neurons innervate the PVH and the DMH (Baquero et al., 2015; Bouret et al., 2004; van der Klaauw et al., 2019). The LHA also receives and send projections to the DMH (Hahn & Swanson, 2010; Thompson & Swanson, 1998) and receive innervation from the arcuate neurons (Bouret et al., 2004; Vogt et al., 2014).

## 5 CONCLUSION

Our study suggests a topographic organization of the projections of the dorsolateral and dorsomedial divisions of the BNST and the adjacent septohypothalamic nucleus onto hypothalamic areas. In agreement with rat data (Dong et al., 2001; Dong & Swanson, 2006) most lateral divisions of the BNST project to lateral areas of the tuberal hypothalamus including the perifornical area and the LHA, while those arising from medial structures such as the dorsomedial BNST and the septohypothalamic nucleus mostly innervate the periventricular including the PVH and the ARH. In particular, the septohypothalamic nucleus projections more intensely innervate the lateral ARH where most of the POMC neurons are observed, than projections arising from the dorsomedial BNST as confirmed by our retrograde study analyses. Collectively, these data suggest a convergent role in feeding and motivated behaviors of these telencephalic structures through projections onto hypothalamic nuclei with potential functional subtleties involving specific neuronal populations.

The anterior BNST is considered as a well‐known stress integrator and is composed of neurons involved in stress and anxiety‐like behaviors (Bowers et al., 2012; Füzesi et al., 2016; Giardino et al., 2018; Khan et al., 2018; Knoll & Carlezon, 2010; Vialou et al., 2014). It receives strong afferences from the amygdala, a well‐described telencephalic structure detecting emotional and biological stressors (Dong et al., 2001; Ip et al., 2019).

Altogether, our data argue in favor of a neuroanatomical basis for the interplay between stress and feeding behavior. However, the molecular and functional characterization of these specific neurocircuits is poorly described and would require further analyses.

## PEER REVIEW

The peer review history for this article is available at https://publons.com/publon/10.1002/cne.24988.

## CONFLICT OF INTEREST

The authors have no conflict of interest to declare.

### Data Availability

The data that support the findings of this study are available from the corresponding author upon reasonable request.
